# Atomic‐Scale Detection of Néel Vector Switching in the Single‐Layer A‐Type Antiferromagnet Cr_2_S_3_‐2D

**DOI:** 10.1002/adma.74637

**Published:** 2026-08-18

**Authors:** Affan Safeer, Calisa Dias, Mahdi Ghorbani‐Asl, Abdallah Karaka, Pradyumna Bawankule, Weibin Li, Pierluigi Gargiani, Wouter Jolie, Arkady V. Krasheninnikov, Amilcar Bedoya‐Pinto, Thomas Michely, Jeison Fischer

**Affiliations:** ^1^ II. Physikalisches Institut Universität zu Köln Köln Germany; ^2^ Institute of Molecular Science University of Valencia Paterna Spain; ^3^ Institute of Ion Beam Physics and Materials Research Helmholtz‐Zentrum Dresden‐Rossendorf Dresden Germany; ^4^ ALBA Synchrotron Light Source Cerdanyola del Vallès Barcelona Spain

**Keywords:** antiferromagnetism, condensed matter physics, graphene, ground state, magnetization, magnetic field, magnetic moment, spintronics, scanning tunneling microscope

## Abstract

The detection of Néel vector switching in a single‐layer A‐type antiferromagnet marks an important step toward functional two‐dimensional spintronics. Here, ${\mathrm{Cr}}_{2}S_{3}$‐2D, grown on graphene on Ir(110), is established as a first single‐layer A‐type antiferromagnet. Spin‐polarized scanning tunneling microscopy reveals hysteresis loops with a large switching field and a pronounced dependence on island size. X‐ray magnetic circular dichroism at the Cr $L_{2,3}$ edges exhibits a tiny signal with a linear magnetic field dependence, consistent with an antiferromagnetic ground state with an estimated net moment below $10^{-2}\mu_{B}$ per Cr and a Néel temperature of about 160 K. Quantitative analysis of the island‐size dependence of the switching field, together with first‐principles calculations, indicates a slight imbalance between the magnetic moments of the two Cr planes of ${\mathrm{Cr}}_{2}S_{3}$‐2D when supported on a substrate. This imbalance results in a net magnetization for the A‐type antiferromagnet, which enables the 180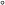 rotation of the Néel vector. Moreover, ${\mathrm{Cr}}_{2}S_{3}$‐2D retains its magnetic properties after several days of exposure to air.

## Graphical abstract

Interfacial electron donation from graphene redistributes charge within single‐layer Cr_2_S_3_‐2D, preferentially accumulating at the lower Cr/S plane. The resulting minute magnetic imbalance lifts the equivalence of the two magnetic sublayers, enabling atomic‐resolution identification and field‐induced control of Néel‐vector states in a two‐dimensional A‐type antiferromagnet.

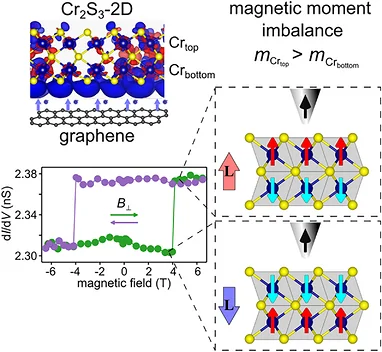

## 1 Introduction

Antiferromagnets (AFs) are emerging as key building blocks for spintronics because they produce no stray field, are robust against external perturbations, and allow ultrafast spin dynamics with the possibility of efficient electrical and optical control [1, 2, 3]. An important aspect of spintronics with AFs is the ability to manipulate and switch the Néel vector, which enables active control of antiferromagnetic order [4, 5]. Extending these advantages to two‐dimensional (2D) materials is particularly appealing, as this promises ultrathin, high‐density spintronic elements and new regimes of low‐dimensional magnetism [6, 7, 8]. In single‐layer AFs, zigzag and stripy orders have been identified [9, 10, 11], but A‐type antiferromagnetic order (Figure 1a)—where ferromagnetic spin layers are antiferromagnetically coupled, providing a layer‐resolved Néel vector for more direct control—has so far only been observed in bilayer van der Waals systems, such as ${\mathrm{CrI}}_{3}$ [12] and CrSBr [13].

**FIGURE 1:**
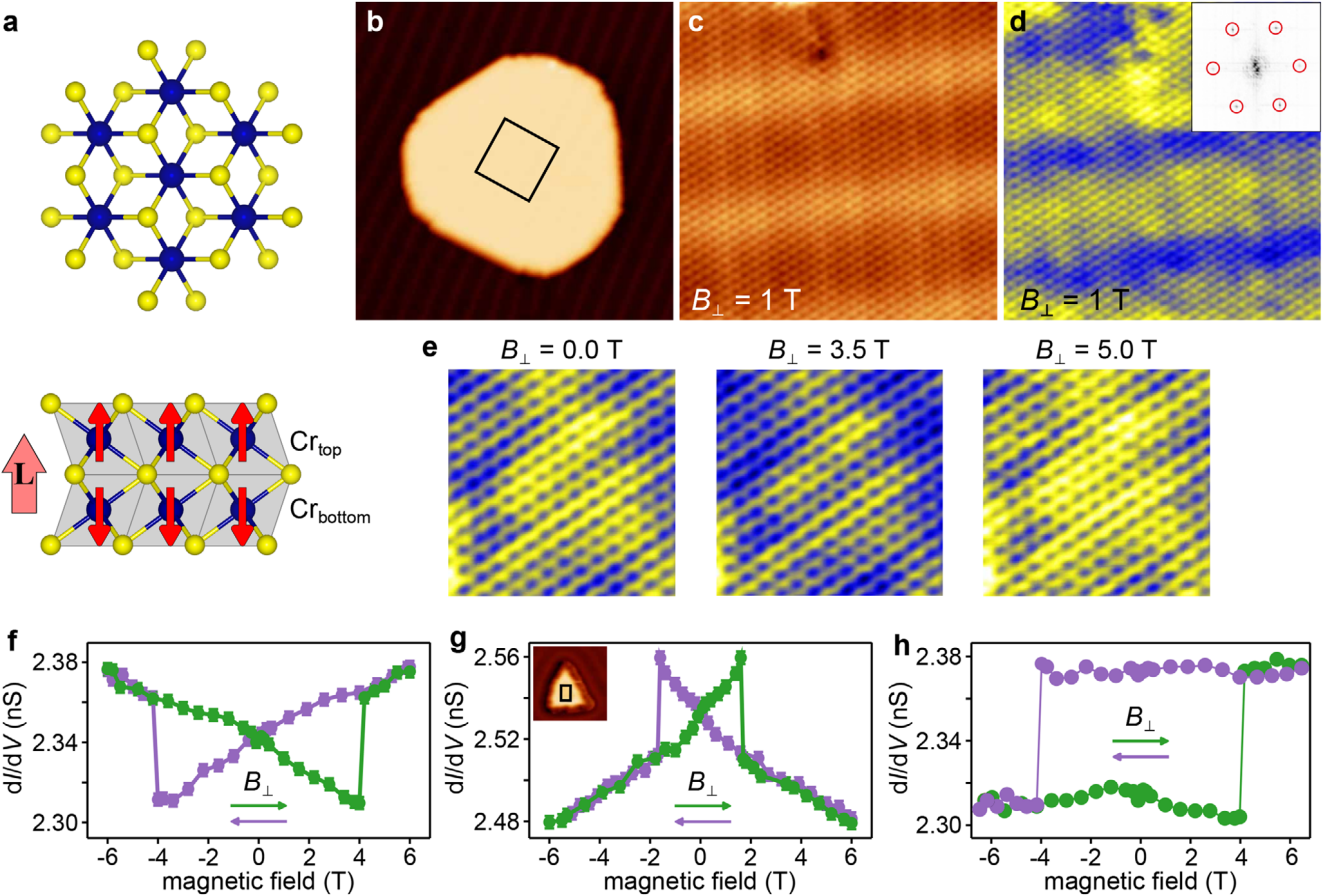
SP‐STM of single‐layer ${\mathrm{Cr}}_{2}S_{3}$‐2D. (a) Top and side view ball models of single‐layer ${\mathrm{Cr}}_{2}S_{3}$‐2D with the two Cr planes labeled ${\mathrm{Cr}}_{\mathrm{top}}$ and ${\mathrm{Cr}}_{\mathrm{bottom}}$. Spins (red arrows) are attached to the Cr atoms in the side view symbolizing A‐type AF order. The Néel vector (**L**) is indicated. Yellow balls: S; blue balls: Cr. (b) STM overview topograph ($V_{b}=1$ V, $I_{t}=20$ pA, 50 nm × 50 nm) of a ${\mathrm{Cr}}_{2}S_{3}$‐2D island on Gr/Ir(110). (c) Atomically resolved SP‐STM topograph ($V_{b}=100$ mV, $I_{t}=1$ nA, 10 nm × 10 nm) of ${\mathrm{Cr}}_{2}S_{3}$‐2D recorded with an out‐of‐plane field of $B_{⊥}=1$ T. (d) dI/dV map of ${\mathrm{Cr}}_{2}S_{3}$‐2D recorded simultaneously with (c). The magnetic contrast of tip was checked independently on a reference sample (Figure S2). The inset shows the fast Fourier transform of the dI/dV map. (e) dI/dV maps ($V_{b}=100$ mV, $I_{t}=1$ nA, 4 nm × 4 nm) recorded at out‐of‐plane fields as indicated. (f) Average dI/dV value as a function of the magnetic field. Each data point is the average over a dI/dV map as in (e). All maps are taken from exactly the same area. (g) Average dI/dV extracted from dI/dV maps taken on a Co island on Cu(111) as a function of magnetic field. The inset shows the dI/dV map ($V_{b}=-300$ mV, $I_{t}=1$ nA, 15 nm × 15 nm) of the Co island on Cu(111), black rectangle shows where data is acquired. (h) De‐convoluted field‐dependent dI/dV response of the ${\mathrm{Cr}}_{2}S_{3}$‐2D, achieved by subtracting the tip contribution established in (g) from (f).

To realize a true single‐layer A‐type AF, we consider the versatile material class of chromium chalcogenides. For example, metastable 1T‐${\mathrm{CrTe}}_{2}$ has emerged as a van der Waals crystal with room‐temperature ferromagnetism [14], whereas in the single‐layer limit it is a zigzag AF with Néel temperature $T_{N}$ ≈ 140 K [10, 15]. In thermodynamic equilibrium, ${\mathrm{CrTe}}_{3}$ is the only chromium chalcogenide van der Waals crystal [16, 17]. The overwhelming majority of chromium chalcogenide compounds are covalently bonded [17, 18, 19]. Therefore, it is not surprising that in recent years several chromium chalcogenide compounds have been synthesized as fully covalently bonded ultrathin films or even 2D materials of single unit cell thickness [15, 20, 21, 22].

Here, we focus on one of these covalently bonded 2D materials, namely ${\mathrm{Cr}}_{2}S_{3}$‐2D, whose structure was recently described [22] and which does not exist in bulk. It can be pictured as five vertically stacked hexagonal planes in a sequence of S–Cr–S–Cr–S as shown in Figure 1a. Each ${\mathrm{Cr}}^{3+}$ ion in ${\mathrm{Cr}}_{2}S_{3}$‐2D is octahedrally coordinated by six S ions with average charge state 2−. Since ${\mathrm{Cr}}_{2}S_{3}$‐2D is different in structure from bulk ${\mathrm{Cr}}_{2}S_{3}$, to avoid confusion, we append “‐2D” to its chemical formula. A comparison of structure and magnetic order for ${\mathrm{Cr}}_{2}S_{3}$‐2D and ‐bulk is provided in Figure S1.

Density functional theory (DFT) calculations for ${\mathrm{Cr}}_{2}S_{3}$‐2D invariably predict ferromagnetic ordering within each Cr plane [22, 23, 24, 25] dominated by in‐plane superexchange with S–Cr–S bond angles close to 90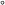. For coupling between the planes, the picture is more complex, and both direct‐exchange‐favoring antiferromagnetic coupling and super‐exchange Cr–S–Cr paths favoring ferromagnetic or antiferromagnetic coupling are present [24]. This ambiguity is reflected in DFT calculations, where the interplane coupling changes from antiferromagnetic to ferromagnetic when the Hubbard U parameter exceeds ≈2 eV [22, 24]. Thus, based on DFT, ${\mathrm{Cr}}_{2}S_{3}$ can be assumed to be an A‐type AF (small U) or a ferromagnet (large U). The latter is assumed by most authors [23, 24, 25].

Recently, two A‐type AFs of the NiAs‐type crystal structure family have been identified as altermagnets [3, 26], namely CrSb [27] and MnTe [28]. If ${\mathrm{Cr}}_{2}S_{3}$‐2D, which is the ultimately thin NiAs‐type 2D material, could be established as A‐type AF, exploring low‐dimensional altermagnetism and its associated transport phenomena would become accessible.

Here, by combining spin‐polarized scanning tunneling microscopy (SP‐STM) with x‐ray magnetic circular dichroism (XMCD) measurements on ${\mathrm{Cr}}_{2}S_{3}$‐2D, we encounter an apparent paradox. While seemingly ferromagnetic hysteresis loops with switching fields of several tesla are observed by SP‐STM, XMCD proves a negligible magnetic moment of the Cr atoms, implying antiferromagnetic order. The puzzle is solved by realizing that switching characterized by a 180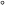 rotation of the Néel vector is enabled by a small uncompensated moment, which arises from the interaction with the substrate.

## 2 Results and Discussion

### 2.1 Hysteresis Loops on ${\mathrm{Cr}}_{2}S_{3}$‐2D Islands

To investigate the magnetic properties of ${\mathrm{Cr}}_{2}S_{3}$‐2D, we obtained at 4.2 K atomically and spin‐resolved STM images together with dI/dV maps of a ${\mathrm{Cr}}_{2}S_{3}$‐2D island, as shown in Figure 1b–e. Both the SP‐STM topograph (Figure 1c) and the corresponding dI/dV map (Figure 1d) obtained at an out‐of‐plane field of 1 T reveal the hexagonal lattice of ${\mathrm{Cr}}_{2}S_{3}$‐2D with a lattice constant of 0.340 nm, superimposed on the moiré pattern of the underlying Gr/Ir(110) substrate (visible as horizontal bright lines in Figure 1c,d). Although at the bias of $V_{b}=100$ mV a magnetic field‐dependent change in the dI/dV signal is observed (see Figure S3), no additional superstructure or magnetic superlattice is detected, as confirmed by the fast Fourier transform of the dI/dV map shown in the inset of Figure 1d.

However, when the magnetic field is varied, the overall signal of the dI/dV maps displays a slight change in contrast (Figure 1e). To systematically probe this behavior, dI/dV maps are acquired over a magnetic field ranging between +6 T and −6 T. To obtain a sufficient signal‐to‐noise ratio, their dI/dV value is averaged in an area of 16 ${\mathrm{nm}}^{2}$ and plotted as a function of magnetic field in Figure 1f. The field‐dependent dI/dV values show a butterfly‐shaped hysteresis, with a quasi‐linear response and symmetrical abrupt jumps at ±4 T.

In SP‐STM, the dI/dV signal depends on the spin polarization of both the STM tip and the sample's top plane [29]. To isolate the tip contribution from the observed hysteresis behavior, after acquiring the field‐dependent dI/dV signal on ${\mathrm{Cr}}_{2}S_{3}$‐2D, the same tip is used to measure a Co island on Cu(111) (inset of Figure 1g), which is a well‐characterized ferromagnet with out‐of‐plane easy axis [30]. The resulting curve exhibits a similar, but inverted butterfly‐shaped hysteresis with abrupt jumps at ±1.6 T (Figure 1g), corresponding to the magnetization switching of the Co island. The gradual change originates from the field‐dependent spin polarization of the STM tip [30, 31]. The butterfly shape is inverted due to the negative spin polarization of the Co islands at the measurement bias [32].

After removing the tip contribution by subtracting the slope extracted from the Co island hysteresis, the resulting field‐dependent dI/dV curve exhibits a square hysteresis (Figure 1h). This result is consistent with long‐range ferromagnetic ordering within the top ${\mathrm{Cr}}_{\mathrm{top}}$ plane, and the abrupt change of dI/dV (in Figure 1f) signifies its reversal. The square shape obtained in our analysis indicates a dominant out‐of‐plane easy axis. This is further corroborated by an unchanged dI/dV signal of the in‐plane field magnetization curve shown in Figure S4.

The switching field is exceptionally large compared to other 2D magnets. For a ferromagnet, such a large switching field is indicative of a strong magnetic anisotropy. However, if the ferromagnetic top ${\mathrm{Cr}}_{\mathrm{top}}$ plane would be antiferromagnetically coupled to the bottom ${\mathrm{Cr}}_{\mathrm{bottom}}$ plane, the large switching field could reflect a small residual net magnetic moment to which the external field couples only weakly.

### 2.2 A‐Type Antiferromagnetic Ground State in ${\mathrm{Cr}}_{2}S_{3}$‐2D

To determine the actual magnetic state, we performed XMCD measurements on single‐layer ${\mathrm{Cr}}_{2}S_{3}$‐2D. XMCD is able to probe both top and bottom Cr planes with high sensitivity in total electron yield, which, in addition, allows element‐specific detection. The XMCD is obtained as the difference between the left ($\mu^{-}$) and the right ($\mu^{+}$) circularly polarized x‐ray absorption spectroscopy (XAS) at the Cr $L_{2,3}$ edges in normal incidence (NI, ϕ = 0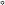) (Figure 2) and grazing incidence (GI, ϕ = 70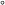) (Figure S5) with the field parallel to the incident beam. These geometries are sensitive to out‐of‐plane and in‐plane spin components, respectively. The XAS exhibits the Cr $L_{3}$ and Cr $L_{2}$ peaks at 575.6 and 583.2 eV with an overall shape consistent with the XAS of bulk chromium sulfide [33]. An STM topograph of the exact sample measured by XMCD is displayed in Figure S5a.

**FIGURE 2:**
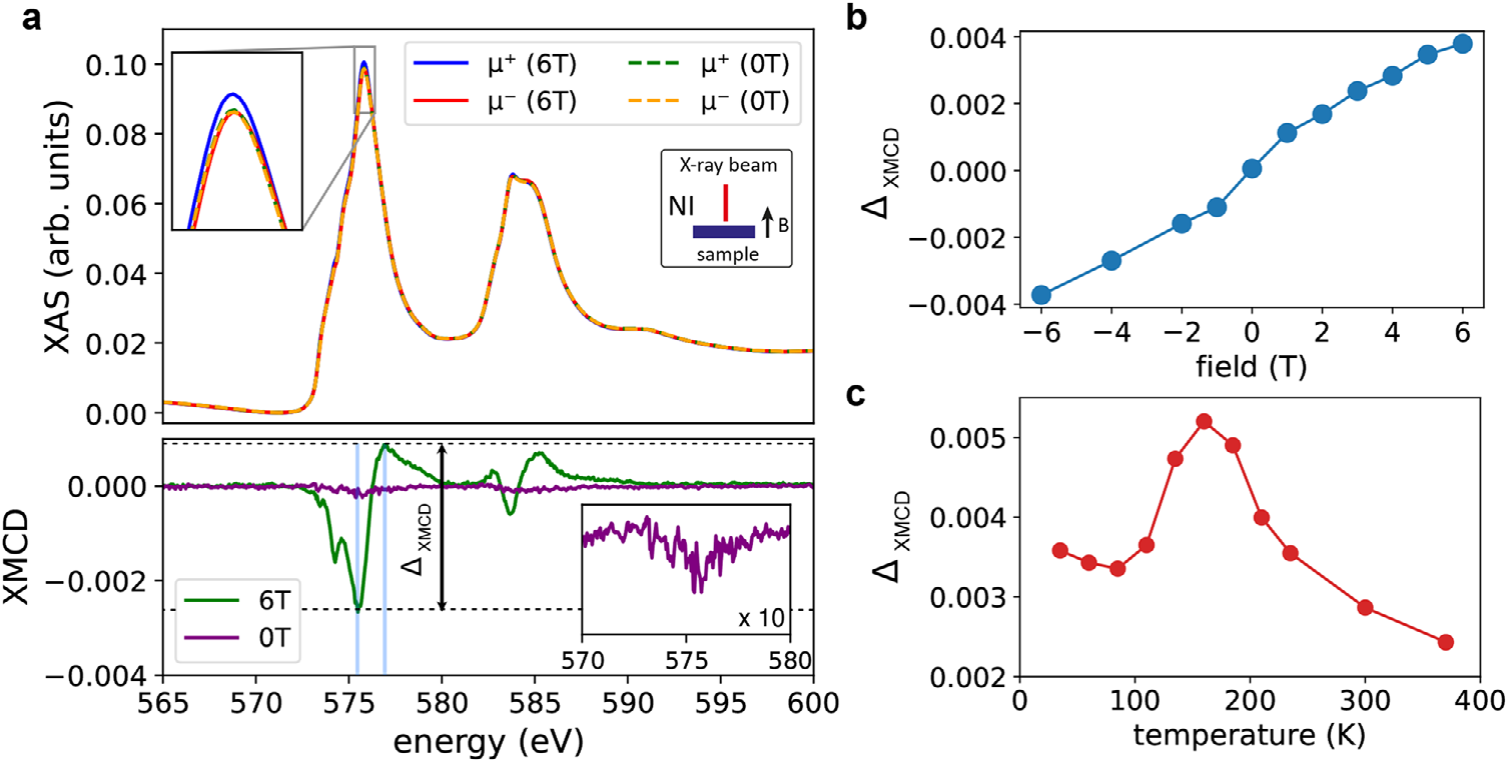
XMCD of single‐layer ${\mathrm{Cr}}_{2}S_{3}$‐2D at normal incidence. (a) Upper panel: Cr $L_{2,3}$ edges XAS of single‐layer ${\mathrm{Cr}}_{2}S_{3}$‐2D on Gr/Ir(110) acquired in total electron yield mode for positive ($\mu^{+}$) and negative ($\mu^{-}$) helicities at 3.5 K with 0 T and 6 T. Insets on the left and right show the zoom of the Cr $L_{3}$ edge and the measurement schematics, respectively. Lower panel: Resulting XMCD signal ($\mu^{-}-\mu^{+}$). Inset shows 0 T XMCD signal multiplied by a factor of 10. (b) Peak‐to‐peak span of XMCD Cr $L_{3}$ edge ($\Delta_{\mathrm{XMCD}}$) as a function of magnetic field at 3.5 K. (c) Temperature‐dependent $\Delta_{\mathrm{XMCD}}$ of Cr $L_{3}$ edge with 6 T applied magnetic field. The $\Delta_{\mathrm{XMCD}}$ values are extracted in both field‐ and temperature‐dependent variation from the values in photon energy marked by lines in the XMCD panel of (a), corresponding to the minimum and maximum signal at the Cr $L_{3}$ edge, and taking the difference (Δ) between them.

The XMCD spectrum in NI (Figure 2a) exhibits a small signal at 6 T. Assuming the validity of XMCD sum rules [34, 35], the estimated magnetic moment per Cr atom is $\leq 0.1\;\mu_{B}$ at 6 T. This small XMCD signal is consistent with field‐induced spin canting of an antiferromagnetic system. Moreover, the nearly linear dependence of the XMCD intensity at the $L_{3}$ edge $\Delta_{\mathrm{XMCD}}$ on the applied magnetic field (Figure 2b) supports an antiferromagnetic ground state, in which the external field enhances the spin canting.

At zero field, the XMCD signal almost vanishes (Figure 2a), but not completely. Such a residual XMCD signal could indicate uncompensated, magnetically correlated spins in ${\mathrm{Cr}}_{2}S_{3}$‐2D. A remanent magnetic signal of all Cr atoms would imply a net magnetic moment per atom $≲5\times 10^{-3}\;\mu_{B}$ at 0 T.

To further clarify the nature of magnetic coupling, we performed temperature‐dependent XMCD measurements for B=6 T (Figure S6). In Figure 2c, we represent $\Delta_{\mathrm{XMCD}}$ showing a non‐monotonic behavior with respect to temperature, reaching a maximum around 160 K above which it decreases steadily.

These results are interpreted as follows: At $T<T_{N}$, strong interplane exchange coupling enforces antiparallel alignment between the Cr planes, thermal excitation progressively weakens this coupling and enables the field‐induced spin canting more easily, resulting in an increase of the XMCD signal. As the temperature approaches $T_{N}$, spin fluctuations and spin flip give rise to a maximum XMCD signal, beyond which the signal decreases as the system becomes paramagnetic. The observed non‐monotonic behavior thus reflects a typical temperature‐dependent susceptibility of an AF, with a transition to a paramagnetic phase at a Néel temperature $T_{N}\approx 160$ K. This Néel temperature is also identified by x‐ray magnetic linear dichroism signal, which drops to zero at $T_{N}$ (Figure S7).

The combination of the ferromagnetic response of the topmost Cr plane observed by SP‐STM (Figure 1) and the compensated antiferromagnetic structure revealed by XMCD (Figure 2) demonstrates that ${\mathrm{Cr}}_{2}S_{3}$‐2D is an A‐type AF.

Although antiferromagnetic order would ideally lead to zero net magnetization, switching as observed in Figure 1 reveals that a magnetization reversal mechanism is available. A spin–flip transition is not expected, as the Zeeman energy (gμB≈1.6 meV per Cr at 5 T) is much smaller than the interlayer exchange energy (51.8 meV per unit cell), obtained from DFT [22]. A more reasonable reversal mechanism is a rotation of the Néel vector by $180^{\circ }$. Switching may arise from uncompensated magnetic moments, typically associated with surface, interface, or edge atoms, which couple to the applied field and induce rotation of the Néel vector. To elucidate the origin of the SP‐STM hysteresis, we carry out measurements for different island sizes.

### 2.3 Size‐Dependent ${\mathrm{Cr}}_{2}S_{3}$‐2D Island Switching

Representative field‐dependent dI/dV curves are presented in Figure 3a–d. The smallest island in Figure 3a exhibits a V‐shaped curve, Figure 3b–d displays butterfly‐shaped $dI/dV(B_{⊥})$ curves as in Figure 1f. The switching field of the islands increases monotonically with their size.

**FIGURE 3:**
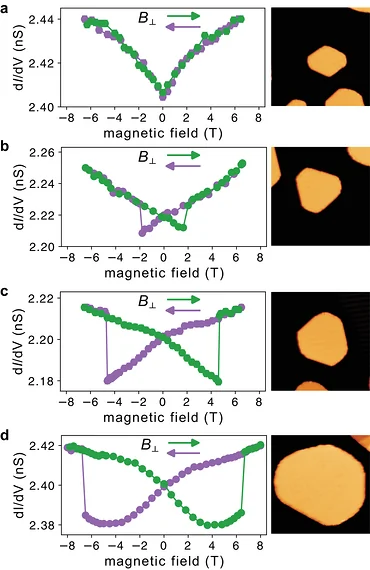
Size‐dependent ${\mathrm{Cr}}_{2}S_{3}$‐2D island switching. (a–d) Average dI/dV ($V_{b}=100$ mV, $I_{t}=1$ nA) as a function of magnetic field (left) and STM topograph ($V_{b}=2$ V, $I_{t}=50$ pA, 60 nm × 60 nm) of corresponding island of measurement (right).

The V‐shaped magnetization curve of Figure 3a signifies that the switching field is zero (the topmost plane behaves like a superparamagnet) or close to zero. Thus, the hysteretic jump in $dI/dV(B_{⊥})$ takes place at zero or close to zero field, that is, it is absent.

The statistical analysis of over 35 islands (Figure 4a) identifies a monotonic increase of the switching field ($\mu_{0}H_{\mathrm{sw}}$) with island size given by its number of Cr atoms ($N_{\mathrm{Cr}}$) for islands larger than $N_{\mathrm{Cr}}\approx$ 6000 atoms (300 ${\mathrm{nm}}^{2}$). Islands smaller than $N_{\mathrm{Cr}}\approx$ 6000 atoms (300 ${\mathrm{nm}}^{2}$) switch near or at zero field.

**FIGURE 4:**
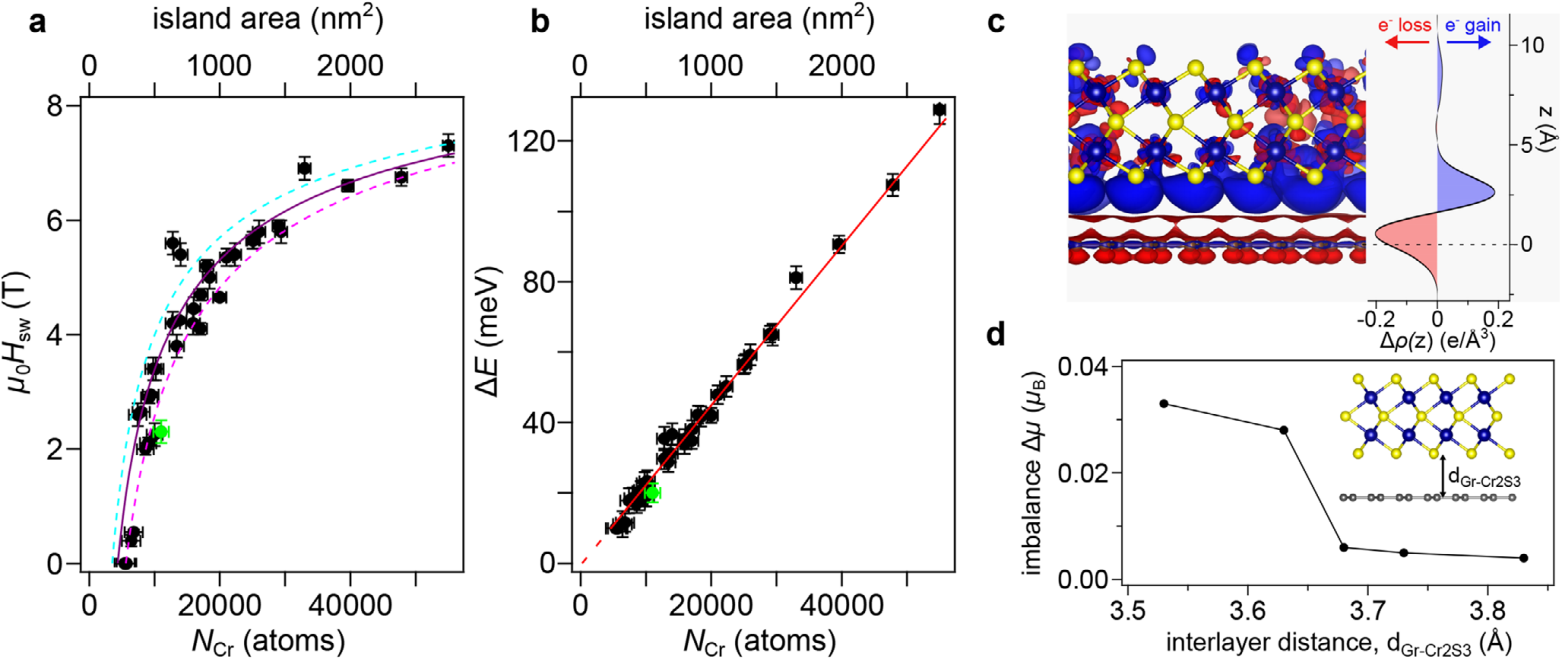
Quantitative estimation of uncompensated moments and charge transfer from substrate. (a) Switching field $\mu_{0}H_{\mathrm{sw}}$ and (b) energy barrier (ΔE) of islands as a function of island size. All data points are acquired with field‐dependent dI/dV maps at 100 mV and 1 nA. Green data points are extractedfrom the air stability measurement presented in Figure 5. ΔE is extracted from $\mu_{0}H_{\mathrm{sw}}$ in (a) by using Equation (1). Violet and red lines in (a) and (b) are fits based on Equation (1) assuming the uncompensated magnetic moment $\Delta \mu =8\times 10^{-3}\;\mu_{B}$ per unit cell to stem from the difference in the Cr atomic moments in the top ${\mathrm{Cr}}_{\mathrm{top}}$ and bottom ${\mathrm{Cr}}_{\mathrm{bottom}}$ planes. Magenta and cyan dashed lines are obtained with smaller $\Delta \mu =6\times 10^{-3}\;\mu_{B}$ and larger $\Delta \mu =1\times 10^{-2}\;\mu_{B}$. (c) Charge density difference plot overlaid with ball model of the relaxed DFT geometries for ${\mathrm{Cr}}_{2}S_{3}$‐2D/Gr. Sphere colors: S: yellow; Cr: blue; C: gray. Charge gain (loss) represented by blue (red). (d) Δμ as a function of distance between Gr and ${\mathrm{Cr}}_{2}S_{3}$‐2D, $d_{\mathrm{Gr}-{\mathrm{Cr}}_{2}S_{3}}$ as indicated in the inset.

### 2.4 The Mechanism of Néel Vector Switching

Several microscopic mechanisms could, in principle, contribute to the field‐induced switching of the top layer magnetization as observed by STM, including antiferromagnetic domain‐wall motion, incomplete reversal of the magnetic state, or coherent rotation of the antiferromagnetic order parameter. Switching by domain‐wall motion or incomplete reversal would imply some stochasticity with respect to location on an island, symmetry of switching for negative and positive fields, island size, or STM setpoint. However, magnitudes of switching fields and $dI/dV(B_{⊥})$ signal do not depend on the tip location on an island (Figure S8), are the same at positive and negative field (Figures 1 and 3), and behave monotonically with island size (Figure 4a) and set point (Figure S9). The absence of significant stochastic variations in the switching behavior suggests a collective reversal of the magnetic state, making coherent rotation of the Néel vector the most consistent interpretation of our observations.

To gain further insight into the reversal mechanism in ${\mathrm{Cr}}_{2}S_{3}$‐2D islands, we extract the energy barrier ΔE from the measured switching fields $\mu_{0}H_{\mathrm{sw}}$. In an antiferromagnetic particle, the dominant contribution to ΔE for a coherent rotation of the Néel vector $L=m_{{\mathrm{Cr}}_{\mathrm{top}}}-m_{{\mathrm{Cr}}_{\mathrm{bottom}}}$ originates from the anisotropy of all Cr atoms, while the applied field couples to a small uncompensated magnetic moment $\mu_{u}$. Here, $m_{{\mathrm{Cr}}_{\mathrm{top}}}$ and $m_{{\mathrm{Cr}}_{\mathrm{bottom}}}$ are the total magnetic moments of the two Cr sublattices.

Thermal fluctuations assist the system in overcoming ΔE; evidence for this statement is that lowering the temperature from 4.2 to 1.7 K increases the switching field (Figure S11). For the case of an uniaxial anisotropy and magnetization reversal, that is, switching of the Néel vector L by $180^{\circ }$ at a temperature T, the relation between the $\mu_{0}H_{\mathrm{sw}}$ and ΔE for a measurement time $t_{\mathrm{meas}}$ is given by a Sharrock‐type expression [36],

(1) $$\mu_{0}H_{\mathrm{sw}}=\frac{2\Delta E}{\mu_{u}}\left[1-{\left(\frac{k_{B}T}{\Delta E}\ln\;\left(\frac{t_{\mathrm{meas}}}{\tau_{0}}\right)\right)}^{1/2}\right],$$

where $k_{B}$ is the Boltzmann constant and $\tau_{0}$ is the attempt time. In our analysis, we use a typical value of $\tau_{0}=10^{-10}\;s$ for the attempt time [37] and $t_{\mathrm{meas}}=100\;s$, corresponding to the duration of the measurement.

To model the total uncompensated magnetic moment $\mu_{u}$ in Equation (1), we assume a difference $\Delta \mu =\mu_{{\mathrm{Cr}}_{\mathrm{top}}}-\mu_{{\mathrm{Cr}}_{\mathrm{bottom}}}$ in the atomic magnetic moments of the two Cr atom planes leading to $\mu_{u}=1/2N_{\mathrm{Cr}}\Delta \mu$ with $N_{\mathrm{Cr}}$ being the total number of Cr atoms in an island ($1/2N_{\mathrm{Cr}}$ in each Cr plane). Assuming a single value Δμ, Equation (1) is solved for each island size characterized by $N_{\mathrm{Cr}}$. Figure 4b shows the energy barrier $\Delta E(N_{\mathrm{Cr}})$ for $\Delta \mu =8\times 10^{-3}\;\mu_{B}$. The data points lie along a linear ΔE dependence on $N_{\mathrm{Cr}}$. This behavior is consistent with an effective Stoner–Wohlfarth‐type description of the antiferromagnetic energy barrier, in which the energy barrier is given by $\Delta E=KN_{\mathrm{Cr}}$, where K is the effective anisotropy energy per Cr atom. The red line in Figure 4b is a linear fit through the data points with the slope being K=2.3μ eV/Cr atom. Replacing in Equation (1) ΔE by $KN_{\mathrm{Cr}}$ and using $\mu_{u}=1/2N_{\mathrm{Cr}}\Delta \mu$, we predict the functional dependence $H_{\mathrm{sw}}\left(N_{\mathrm{Cr}}\right)$ shown as violet line in Figure 4a. It is evident that the choice of $\Delta \mu =8\times 10^{-3}\;\mu_{B}$ provides a decent fit to the experimental data. Smaller ($\Delta \mu =6\times 10^{-3}\;\mu_{B}$, magenta dashed line) and larger ($\Delta \mu =1\times 10^{-2}\;\mu_{B}$, cyan dashed line) values of Δμ provide inferior fits. We emphasize that Δμ is the only fit parameter in the entire procedure defining $\mu_{u},\Delta E,K$, and $H_{\mathrm{sw}}\left(N_{\mathrm{Cr}}\right)$. Recalculating Δμ into a fictitious effective magnetic moment per Cr atom yields a value of $4\times 10^{-3}\;\mu_{B}$ per atom, which is consistent with our rough estimate from XMCD at 0 T. As an alternative model for $\mu_{u}$ in Equation (1), we tested the assumption that it stems from the uncompensated spins of the Cr island edge atoms and assigned to each edge atom a magnetic moment $\mu_{e}$. As documented by Figure S11, this model is clearly worse than the one of Figure 4. Edge effects are not dominant for switching of islands.

Interface‐induced magnetic effects are well established and include phenomena such as proximity‐induced magnetization, spin–orbit coupling driven surface and interface effects, and magnetism arising from charge transfer [38]. The latter is well known for oxide superlattices [39, 40]. In 2D heterostructures, charge transfer has also been shown to modify interplane exchange interactions [41]. For ${\mathrm{Cr}}_{2}S_{3}$‐2D, charge transfer from the substrate is inferred from the fact that ab initio calculations consistently show freestanding ${\mathrm{Cr}}_{2}S_{3}$ to be semiconducting [22, 23, 24, 25], whereas when grown on Gr/Ir(110), it is metallic [22]. The electron transfer from the substrate into the ${\mathrm{Cr}}_{2}S_{3}$‐2D conduction band is accompanied by the formation of an interface dipole, which originates from vacuum level alignment [22].

The charge transfer from Gr to ${\mathrm{Cr}}_{2}S_{3}$‐2D and the resulting imbalance in the magnetic moments are captured by DFT calculations. We employed a DFT supercell (Figure S12) of a 5×5 ${\mathrm{Cr}}_{2}S_{3}$‐2D layer matching a 7×7 Gr layer almost free of strain. Upon structural relaxation, the interlayer distance converges to $d_{\mathrm{Gr}-{\mathrm{Cr}}_{2}S_{3}}=3.53$ Å. At this separation, charge transfer occurs from Gr to ${\mathrm{Cr}}_{2}S_{3}$‐2D, leading to charge accumulation at the bottom S plane, and a corresponding depletion on the Gr side, as illustrated in Figure 4c. Without Gr (i.e., freestanding ${\mathrm{Cr}}_{2}S_{3}$‐2D), we find an average magnetic moment of 2.79 $\mu_{B}$ per Cr atom in each Cr plane. However, with Gr underneath, because the interfacial charge redistribution modifies the occupation of the spin‐polarized states in ${\mathrm{Cr}}_{2}S_{3}$‐2D, the average magnetic moment in the bottom ${\mathrm{Cr}}_{\mathrm{bottom}}$ plane decreases to 2.77 $\mu_{B}$ per Cr atom, while the top ${\mathrm{Cr}}_{\mathrm{top}}$ plane exhibits a magnetic moment of 2.80 $\mu_{B}$ (Figure S12). The small imbalance $\Delta \mu =0.03\;\mu_{B}$ per unit cell ($\Delta \mu =0.015\;\mu_{B}$/Cr) agrees qualitatively with the estimate $\Delta \mu =0.008\;\mu_{B}$ per unit cell ($\Delta \mu =0.004\;\mu_{B}$/Cr) based on our model. The quantitative discrepancy is not unexpected, as the DFT calculations do not explicitly include the Ir substrate, and the effective moment entering the Sharrock analysis may differ from the static local moment obtained from DFT due to dynamical and screening effects. Increasing the distance between Gr and ${\mathrm{Cr}}_{2}S_{3}$‐2D reduces Δμ, restoring the magnetic moments to values close to those of freestanding ${\mathrm{Cr}}_{2}S_{3}$‐2D.

Taking a closer look at the fit curve in Figure 4a, we find that not only the size dependence of $\mu_{0}H_{\mathrm{sw}}$ but also the onset of switching at a finite island size is well reproduced. Small islands are not in a blocked state, because $T>\frac{KN}{25k_{B}}$ [42].

An additional observation is that the switching behavior exhibits a dependence on the tunneling set point during spectroscopy mapping (Figure S9). Variation of the tunneling set point changes the tip electric field, the Joule heating, or even the spin transfer torque. Irrespective of the precise origin, the effect on the $\mu_{0}H_{\mathrm{sw}}$ collection in Figure 4a is minor since all the measurements were at 1 nA and extrapolating to zero current yields a less than 4% larger switching field, still within the measurement error bars. However, a large current of 7 nA decreases the switching field by nearly 1 T (Figure S9), providing an alternative route to control the Néel vector switching with lower fields.

Our analysis suggests that the ${\mathrm{Cr}}_{2}S_{3}$‐2D islands can be regarded as an effective ferrimagnetic system, in which the top and bottom Cr plane sublattices have a slight difference in magnetic moment, actively driving the reversal process. While responding to the field via the Zeeman term, the uncompensated moments allow a field‐driven rotation of the Néel vector L by $180^{\circ }$. Because SP‐STM is a surface‐sensitive technique, an inversion of the L in this A‐type AF is readily detected through the change of magnetization in the top ${\mathrm{Cr}}_{\mathrm{top}}$ plane as in Figures 1 and 3. This picture provides a first‐order description that captures the key features of thermally activated Néel vector reversal.

### 2.5 Magnetic Order in ${\mathrm{Cr}}_{2}S_{3}$‐2D is Air‐Stable

To examine the robustness of the new 2D antiferromagnet, we exposed the sample to ambient conditions for 2 days. The STM image of Figure 5a acquired after reintroducing the sample to ultrahigh vacuum (UHV) without any additional treatment shows intact ${\mathrm{Cr}}_{2}S_{3}$‐2D islands with preserved morphology. However, adsorbates are present on both graphene and ${\mathrm{Cr}}_{2}S_{3}$‐2D, impairing SP‐STM measurements. To remove these adsorbates, the sample is briefly annealed to 600 K in UHV. As shown in Figure 5b, while adsorbates remain at edges and grain boundaries, they desorb from the island area and leave its surface largely clean. Subsequent $dI/dV(B_{⊥})$ measurements (Figure 5c) reproduce the characteristic hysteresis observed prior to air exposure. The inset of Figure 5c displays the measured island containing ≈11000 Cr atoms. The $\mu_{0}H_{\mathrm{sw}}\left(N_{\mathrm{Cr}}\right)$ data point is plotted as a green dot in Figure 4. It fits to the trend demonstrating that exposure to air does not significantly affect switching. The persistence of the hysteresis confirms that the magnetic ground state of single‐layer ${\mathrm{Cr}}_{2}S_{3}$‐2D remains unchanged after prolonged exposure to ambient conditions.

**FIGURE 5:**
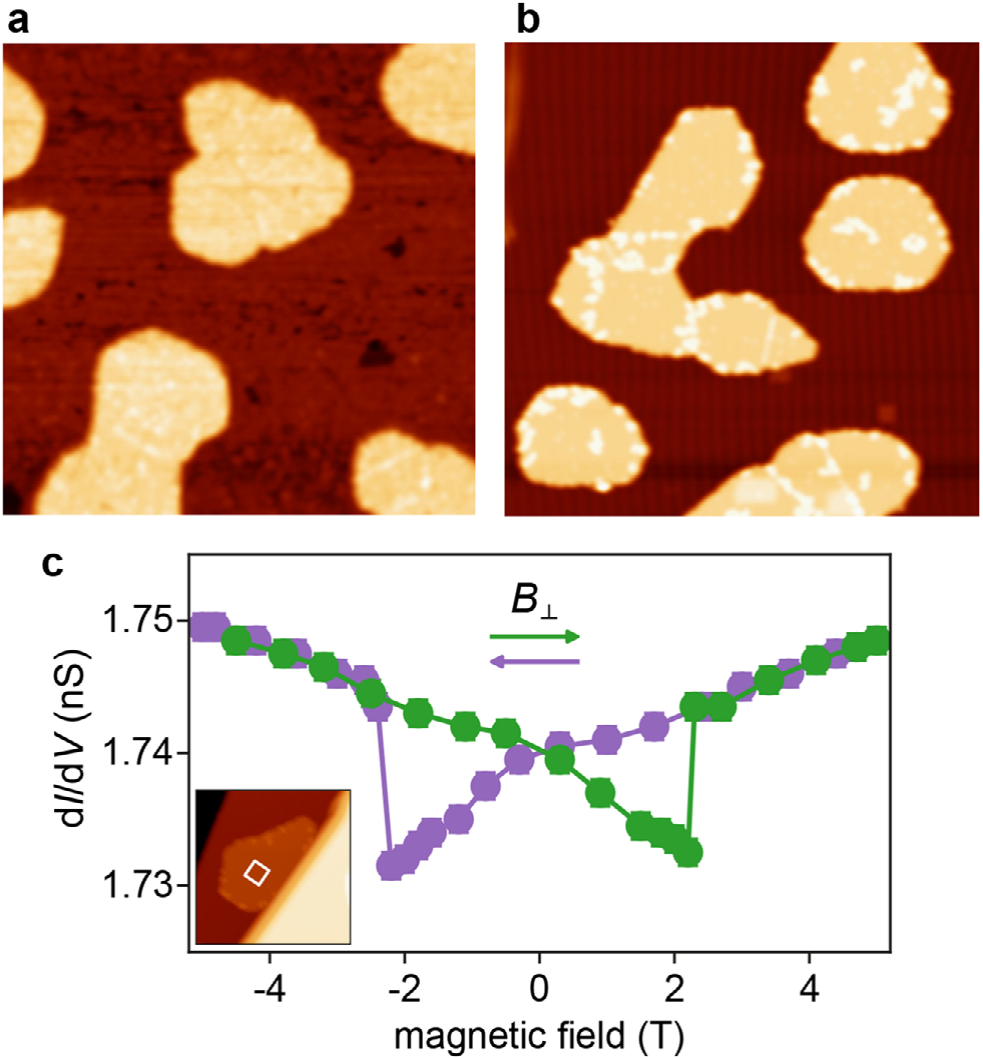
Air stability of single‐layer ${\mathrm{Cr}}_{2}S_{3}$‐2D. (a) STM topograph ($V_{b}=2$ V, $I_{t}=50$ pA, 120 nm × 120 nm) of ${\mathrm{Cr}}_{2}S_{3}$‐2D on Gr/Ir(110) taken after exposure to air for 2 days and subsequent reintroduction to UHV without any additional treatment. (b) STM topograph ($V_{b}=1$ V, $I_{t}=50$ pA, 120 nm × 120 nm) of the same sample after annealing at 600 K for 60 s. (c) Averaged dI/dV signal as a function of magnetic field. Inset shows the STM topograph ($V_{b}=2$ V, $I_{t}=50$ pA, 50 nm × 50 nm) of the island (white square) where field dependence dI/dV measurements are carried out.

## 3 Conclusions

In summary, small ${\mathrm{Cr}}_{2}S_{3}$‐2D islands display unblocked behavior in SP‐STM at 4.2 K, and above a size of $6\times 10^{3}$ Cr atoms islands exhibit a clear hysteresis. XMCD at 3.5 K and the Cr $L_{2,3}$ edges exhibit only a small signal for 6 T, of which only a small fraction is left at remanence (0 T). The overall XMCD response shows a linear dependence on the external magnetic field and a non‐monotonic temperature dependence, aligning with nearly compensated antiferromagnetic order and an ordering temperature of about 160 K. Exposure of ${\mathrm{Cr}}_{2}S_{3}$‐2D to the ambient does not change its magnetic response. Thus, ${\mathrm{Cr}}_{2}S_{3}$‐2D is an air‐stable single‐layer A‐type AF with a Néel temperature of 160 K.

Using a Sharrock‐type expression for the dependence of the switching field on the effective island moment and the energy barrier for 180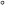 Néel vector rotation, quantitative agreement with the experimental data is obtained for a tiny imbalance in top and bottom plane Cr atom moments. According to our DFT calculations, the origin of the imbalance in magnetic moments is a charge transfer from the graphene substrate primarily to the bottom ${\mathrm{Cr}}_{\mathrm{bottom}}$ atom plane of ${\mathrm{Cr}}_{2}S_{3}$‐2D.

Our research implies that for a fixed external magnetic field the Néel vector in ${\mathrm{Cr}}_{2}S_{3}$‐2D can be controlled by gates. A promising device architecture would be a ${\mathrm{Cr}}_{2}S_{3}$‐2D/Gr heterostructure encapsulated in h‐BN. In this configuration, electrostatic gating could modulate the graphene‐to‐${\mathrm{Cr}}_{2}S_{3}$‐2D charge transfer, analogous to the STM‐tip‐induced tuning shown in Figure S9, thereby providing direct control over the Néel‐vector switching characteristics. Being air stable and transferable, only bound by van der Waals forces to Gr, ${\mathrm{Cr}}_{2}S_{3}$‐2D possesses the essential properties for electrical switching, paving the way for future device‐oriented experiments.

## 4 Experimental Section

### 4.1 Sample Preparation

${\mathrm{Cr}}_{2}S_{3}$‐2D on Gr/Ir(110) is grown in Cologne in an UHV chamber (base pressure = $1\times 10^{-10}$ mbar), enabling UHV transfer into the SP‐STM system. To grow Gr on Ir(110), it is first cleaned and prepared in its unreconstructed state using cycles of 1 keV ${\mathrm{Ar}}^{+}$ sputtering, flash annealing to 1510 K, and subsequent cooling to 400 K in $1\times 10^{-7}$ mbar oxygen pressure. Afterward, Ir(110) is heated to 1510 K and then exposed to $3\times 10^{-7}$ mbar ethylene for 240 s resulting in single crystal Gr on Ir(110) [43]. To grow single‐layer ${\mathrm{Cr}}_{2}S_{3}$‐2D, Gr/Ir(110) is exposed to a Cr flux of $5\times 10^{16}$ atoms $m^{-2}$ $s^{-1}$ from an e‐beam evaporator for 300 s in a sulfur background pressure of $5\times 10^{-8}$ mbar measured by a distant ion gauge. The S pressure is obtained by decomposing pyrite (${\mathrm{FeS}}_{2}$) in a Knudsen cell. After growth, the sample is subsequently annealed at 850 K for 300 s in the presence of a S background pressure of $5\times 10^{-8}$ mbar [22].

### 4.2 STM and SP‐STM Measurements

STM measurements are performed at 4.2 K. Constant‐current topographies and dI/dV maps were recorded with sample bias $V_{b}$ and tunneling current $I_{t}$, as detailed in the respective figure captions. The lock‐in technique with a modulation voltage of 20 mV at 667 Hz is used to record the dI/dV maps. The average dI/dV value from the dI/dV maps is extracted by using Gwyddion software [44]. Spin‐polarized STM tips are obtained by dipping a clean tungsten tip into a ${\mathrm{Cr}}_{2}S_{3}$‐2D island, followed by subsequent bias pulsing on Gr/Ir(110) areas. In between measurements the magnetic contrast of the tips were checked on bilayer Fe nanoisland on Cu(111), as exemplified in Figure S2, where it provides the expected helical magnetic order in the Fe island [45].

### 4.3 XMCD Measurements

XMCD measurements were performed at the BOREAS beamline at ALBA Synchrotron with the collaboration of ALBA staff [46]. The XMCD measurements were performed in total electron yield (TEY) mode, measuring the sample drain current to ground. The signal was normalized to the incoming photon flux measured as the drain current signal on a frshly evaporated gold mesh placed between the last optical element and the sample. The TEY signal surface sensitivity is determined by the secondary electron escape depth and is of the order of several nanometers in the Cr $L_{2,3}$ energy range. The samples were transported from Cologne to Barcelona, either stored in a UHV suitcase ($5\times 10^{-10}$ mbar) or an Ar‐filled container. In both cases, the samples were mounted to XMCD sample holders in an Ar glove box directly connected to the XMCD UHV chamber. Finally, prior to XMCD measurements, the samples were annealed to 600 K in $5\times 10^{-8}$ mbar S for 300 s in a UHV preparation chamber directly connected to the XMCD facility. The XMCD results obtained did not depend on the method of sample transport.

### 4.4 DFT Calculations

The energetics and magnetic properties of all considered systems were investigated using spin‐polarized density functional theory (DFT) as implemented in the VASP code [47, 48]. All the calculations were performed using the Perdew–Burke–Ernzerhof exchange‐correlation functional [49]. A plane‐wave cut‐off energy of 500 eV and force tolerance of 0.01 eV/Å were set for geometry optimization. Van der Waals interactions were included using the DFT‐D2 method proposed by Grimme [50]. A vacuum space of approximately 30 Å was introduced along the non‐periodic direction to avoid interactions between periodic images. The ${\mathrm{Cr}}_{2}S_{3}$‐2D on graphene heterostructure was constructed by using an interface consisting of (5 × 5) unit cells of ${\mathrm{Cr}}_{2}S_{3}$‐2D and (7 × 7) unit cells of graphene, corresponding to a lattice mismatch of only 0.23%. The Brillouin zone of the supercells was sampled using 4 × 4 k‐points mesh. Charge transfer from graphene to ${\mathrm{Cr}}_{2}S_{3}$‐2D was calculated as follows:

(2) $$\Delta \rho \left(z\right)=\rho_{\mathrm{hetero}}\left(z\right)-\left[\rho_{\mathrm{graphene}}\left(z\right)+\rho_{{\mathrm{Cr}}_{2}S_{3}}\left(z\right)\right],$$

where $\rho_{\mathrm{hetero}}$ is the charge density of the heterostructure, $\rho_{\mathrm{graphene}}$ represents the charge density of isolated graphene, and $\rho_{{\mathrm{Cr}}_{2}S_{3}}$ denotes the charge density of ${\mathrm{Cr}}_{2}S_{3}$‐2D. The charge density difference was visualized using the VESTA package [51].

## Conflicts of Interest

The authors declare no conflicts of interest.

## Supporting information


**Supporting File**: adma74637‐sup‐0001‐SuppMat.pdf.

## Data Availability

The data that support the findings of this study are available from the corresponding author upon reasonable request.
