# Supplementary material for: Atomic‐Scale Detection of Néel Vector Switching in the Single‐Layer A‐Type Antiferromagnet Cr2S3‐2D
Source: Adv Mater. 2026 Aug 18;38(58):e74637. doi: 10.1002/adma.74637 (PMC13674632; doi:10.1002/adma.74637)
Supplement: Supplementary file 1 [file ADMA-38-e74637-s001.pdf]

Supplementary Information:
Atomic-Scale Detection of Néel Vector Switching in the Single-Layer A-type Antiferromagnet Cr_2S_3-2D

Affan Safeer,[1,*] Calisa Dias,[2] Mahdi Ghorbani-Asl,[3] Abdallah Karaka,[1] Pradyumna Bawankule,[2] Weibin Li,[4] Pierluigi Gargiani,[4] Wouter Jolie,[1] Arkady V. Krasheninnikov,[3] Amilcar Bedoya-Pinto,[2] Thomas Michely,[1] and Jeison Fischer[1,†]

[1] *II. Physikalisches Institut, Universität zu Köln, Zülpicher Str. 77, 50937 Cologne, Germany*

[2] *Institute of Molecular Science, University of Valencia, Catedratico Jose Beltrán 2, 46980 Paterna, Spain*

[3] *Institute of Ion Beam Physics and Materials Research, Helmholtz-Zentrum Dresden-Rossendorf, 01328 Dresden, Germany*

[4] *ALBA Synchrotron Light Source, Cerdanyola del Vallès, 08290 Barcelona, Spain*

[*] safeer@ph2.uni-koeln.de

[†] jfischer@ph2.uni-koeln.de

CONTENTS

Supplementary Note 1. Comparison of Cr_2S_3-bulk and Cr_2S_3-2D atomic and magnetic structures 3
Supplementary Note 2. Spin-polarized STM tip characterization on an Fe island on Cu(111). 4
Supplementary Note 3. Spin-polarized dI/dV spectra of single-layer Cr_2S_3-2D. 5
Supplementary Note 4. Average dI/dV signal as a function of in-plane and out-of-plane magnetic field. 6
Supplementary Note 5. XMCD of single-layer Cr_2S_3-2D on Gr/Ir(110) for grazing incidence. 7
Supplementary Note 6. Temperature-dependent XMCD of Cr_2S_3-2D. 8
Supplementary Note 7. Temperature-dependent XLD of Cr_2S_3-2D. 9
Supplementary Note 8. Location independence hysteresis behavior of dI/dV signal 10
Supplementary Note 9. Dependence of switching field on tunneling current 11
Supplementary Note 10. Temperature-dependent SP-STM of Cr_2S_3-2D. 12
Supplementary Note 11. Energy barrier analysis with uncompensated moments arising from edges 13
Supplementary Note 12. DFT supercell Cr_2S_3-2D / Gr and comparison to freestanding Cr_2S_3-2D 14

References 14

Supplementary Note 1. Comparison of Cr_2S_3-bulk and Cr_2S_3-2D atomic and magnetic structures

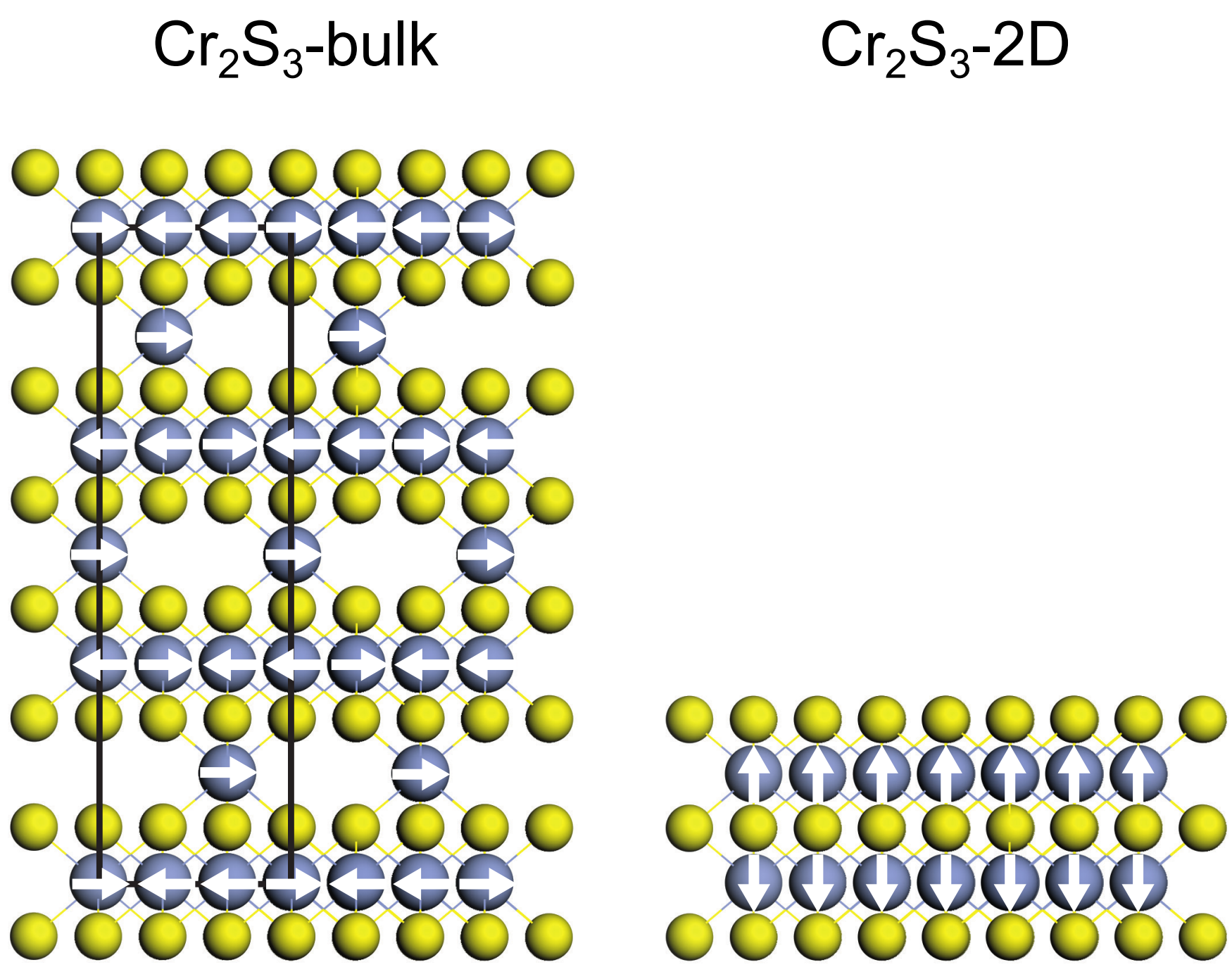

Figure S1. Schematic side view ball models of Cr_2S_3-bulk and Cr_2S_3-2D. Magnetic moments on Cr atoms are indicated by white arrows. Cr_2S_3-bulk is rhombohedral [1], and it has ferrimagnetic order with three sublattices and in-plane anisotropy [2]. The recently described Cr_2S_3-2D structure differs from the bulk [3]. The magnetic order outlined for Cr_2S_3-2D is the topic of the main paper.

Supplementary Note 2. Spin-polarized STM tip characterization on an Fe island on Cu(111).

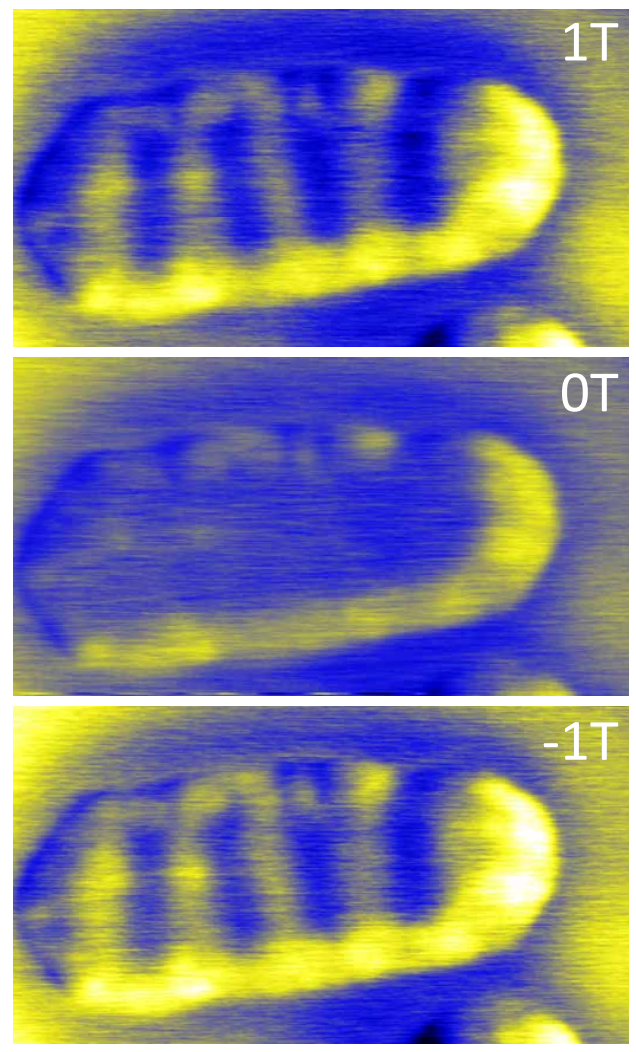

Figure S2. Constant height dI/dV map (V_b = $-400\,\mathrm{mV}$, I_t = $3\,\mathrm{nA}$, $15\,\mathrm{nm} \times 7.5\,\mathrm{nm}$) of an Fe island on Cu(111) acquired at the magnetic fields indicated. To establish the magnetic tip properties independently, they were checked on bilayer Fe islands grown on Cu(111). A tip giving rise to dI/dV maps exhibiting the one-dimensional helical spin order in the Fe islands as described in the literature [4] was considered as suitable.

Supplementary Note 3. Spin-polarized dI/dV spectra of single-layer Cr_2S_3-2D.

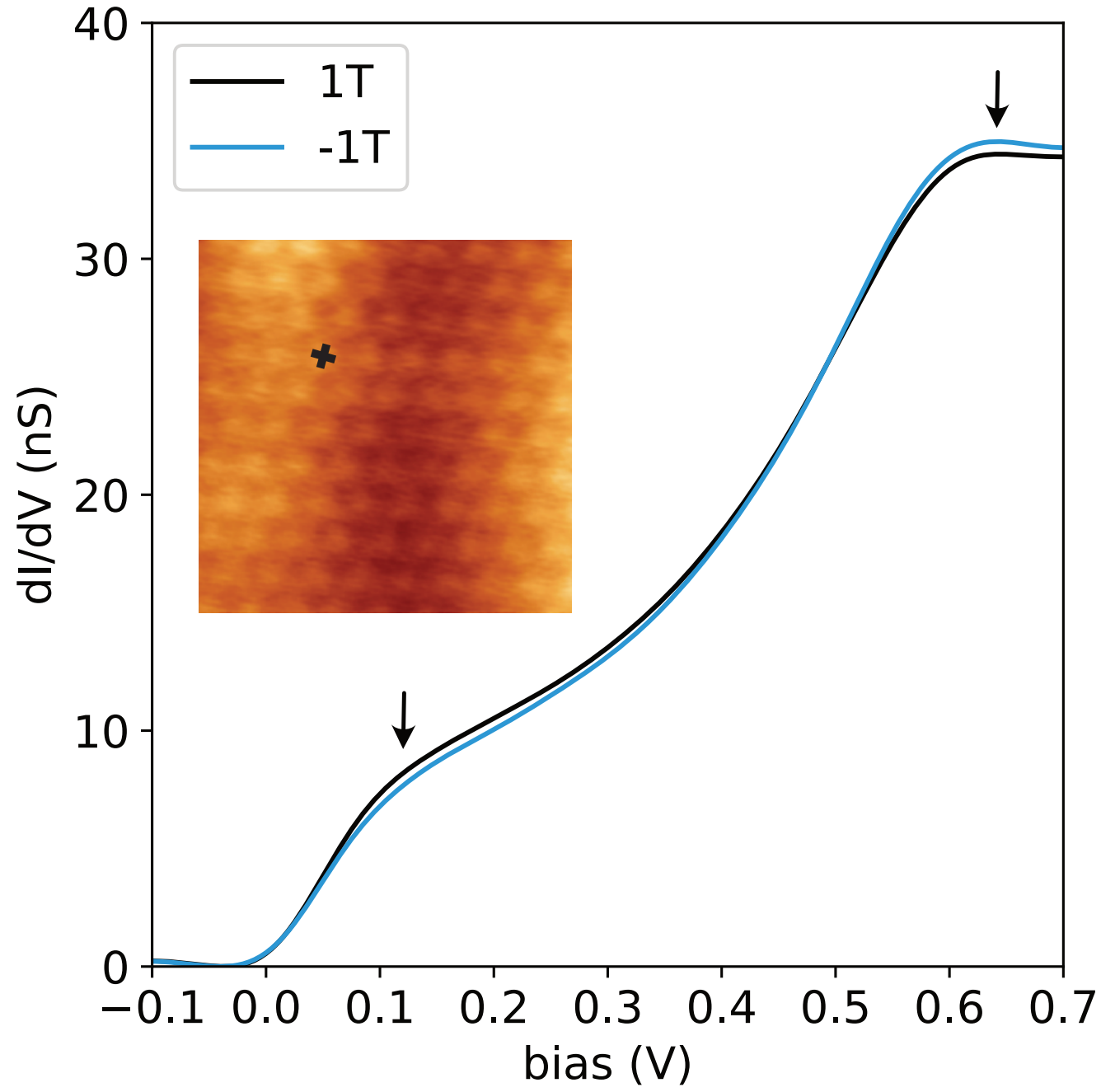

Figure S3. Constant height spin polarized dI/dV point spectra of Cr_2S_3-2D, taken at 1 T and −1 T. The inset shows the atomically resolved STM image (V_b = 100 mV, I_t = 1 nA) of Cr_2S_3-2D, with a black cross indicating the location where spectra are acquired. The black arrows point to dI/d$V(V)$ locations with finite spin polarization. The spectra are acquired by stabilizing at V_b = 700 mV with setpoint current I_t = 1 nA. STM image size = 4 nm × 4 nm.

Supplementary Note 4. Average dI/dV signal as a function of in-plane and out-of-plane magnetic field.

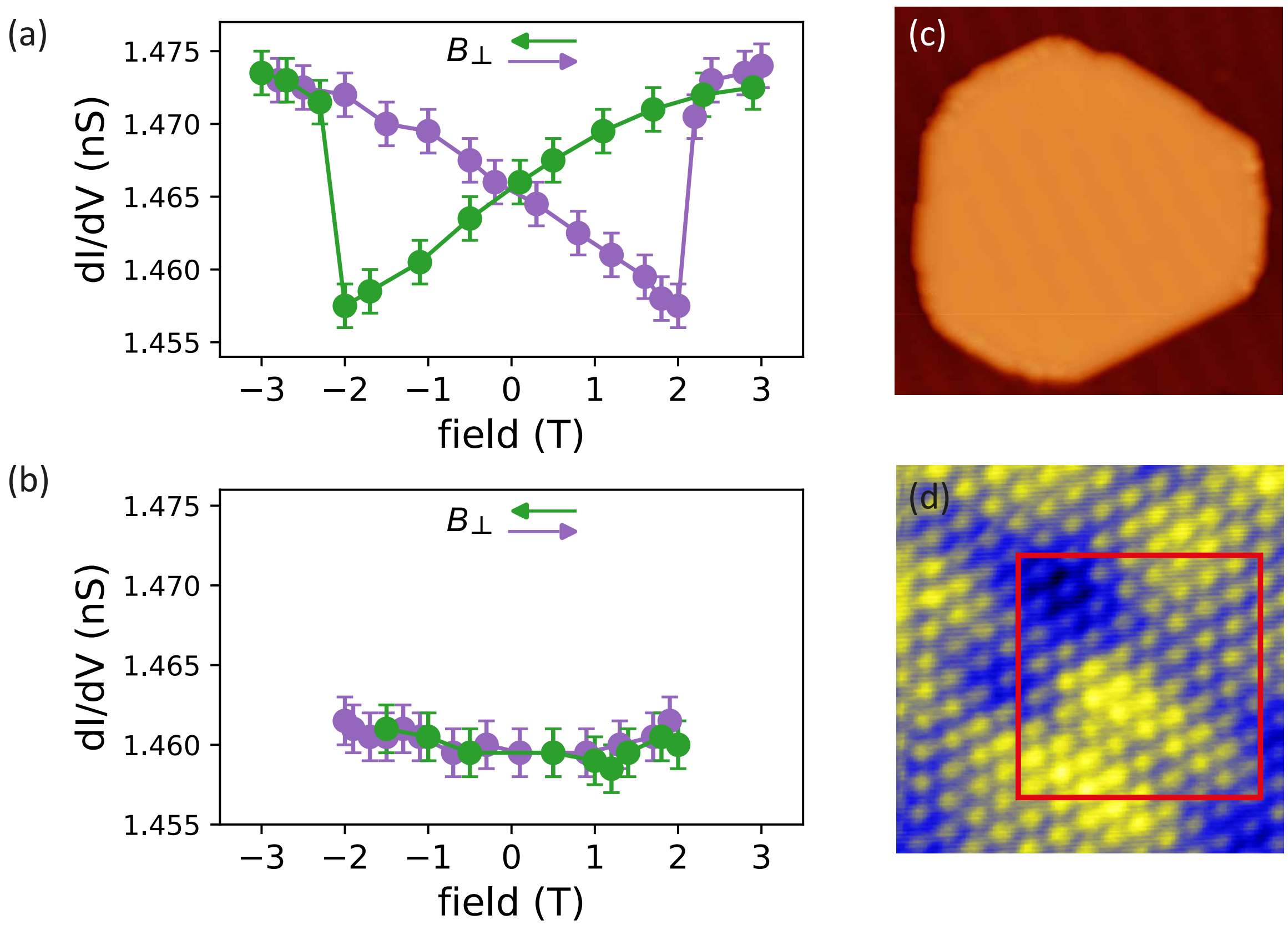

Figure S4. Average dI/dV plots as a function of (a) out-of-plane and (b) in-plane applied magnetic field. (c) STM topograph (V_b = 100 mV, I_t = 1 nA, 32 nm × 32 nm) of the Cr_2S_3-2D island (size ≈ 480 nm^2) on which data is acquired. (d) Constant current dI/dV map (V_b = 100 mV, I_t = 1 nA, 4 nm × 4 nm) of the island shown in (a). The red box in (d) indicates the area used for averaging the dI/dV signal at different magnetic fields.

Supplementary Note 5. XMCD of single-layer Cr_2S_3-2D on Gr/Ir(110) for grazing incidence.

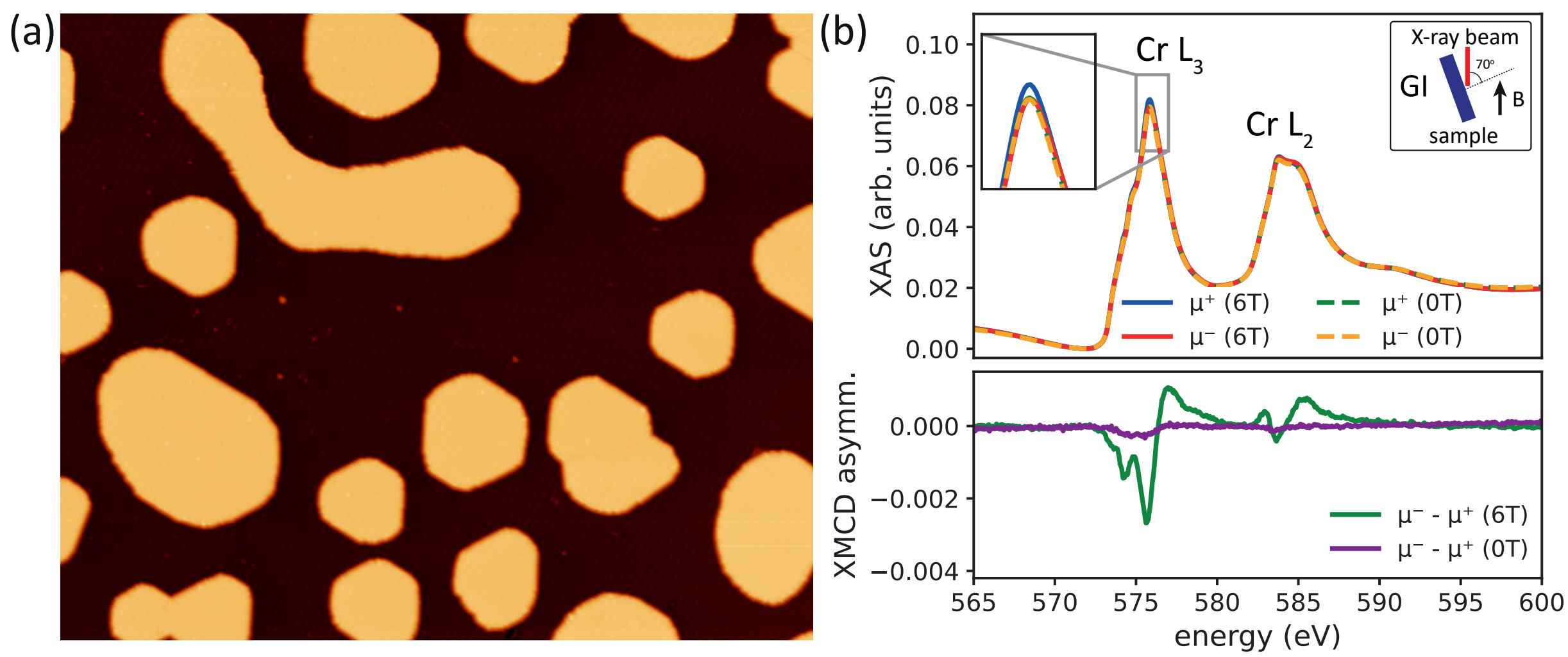

Figure S5. (a) STM topograph (V_b = 1 V, I_t = 50 pA, 300 nm × 250 nm) of Cr_2S_3 sample used for XMCD measurement, which is transferred to BOREAS beamline at ALBA Synchrotron (details given in experimental section of main) (b) Cr $L_{2,3}$ edges XAS and XMCD spectra of single-layer Cr_2S_3-2D on Gr/Ir(110) acquired in total electron yield mode with grazing incidence at 3.5 K under 6 T and 0 T magnetic field. Insets on the left and right show the zoom of the Cr L_3 edge and the measurement schematics, respectively. The XMCD spectrum is obtained by subtracting μ^- from μ^+ XAS. For an AF with out-of-plane anisotropy the required field B_{sat} to saturate the magnetization of both Cr planes to an in-plane direction is given by the relation of exchange energy and the size of the magnetic moment: $B_{sat} = \frac{E_{AFM}-E_{FM}}{\mu}$. With $\mu = 2.8\ \mu_B$ one obtains B_{sat} = 320 T. The resulting canting due to the external field can be estimated with $\theta = \arcsin \frac{B}{B_{sat}} \approx 1°$. Therefore, the observed small signal in grazing incidence XMCD is consistent with an AF with out-of-plane anisotropy.

Supplementary Note 6. Temperature-dependent XMCD of Cr_2S_3-2D.

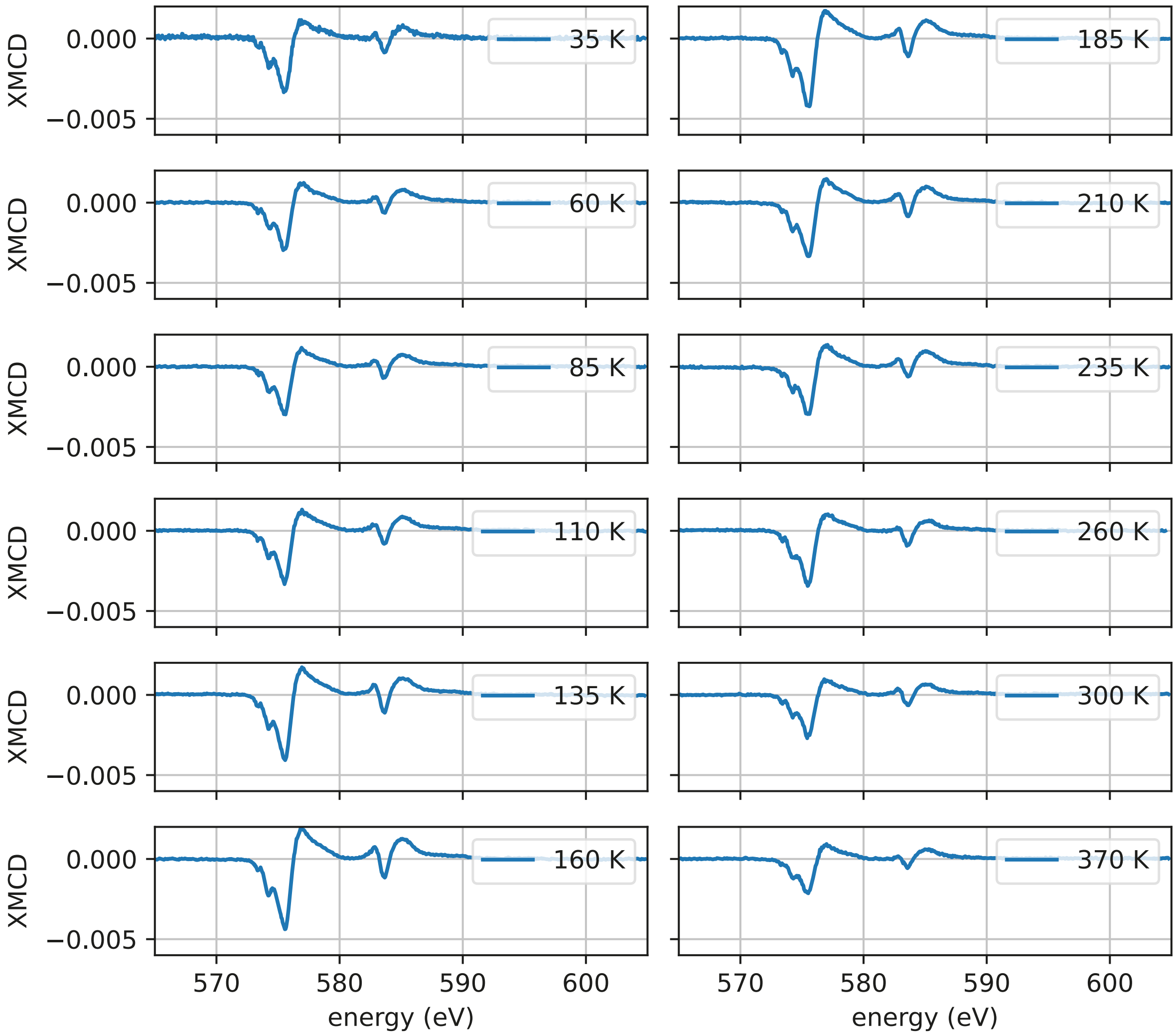

Figure S6. XMCD spectra of single-layer Cr_2S_3-2D as a function of temperature, acquired with the NI X-ray beam at 6 T magnetic field.

Supplementary Note 7. Temperature-dependent XLD of Cr_2S_3-2D.

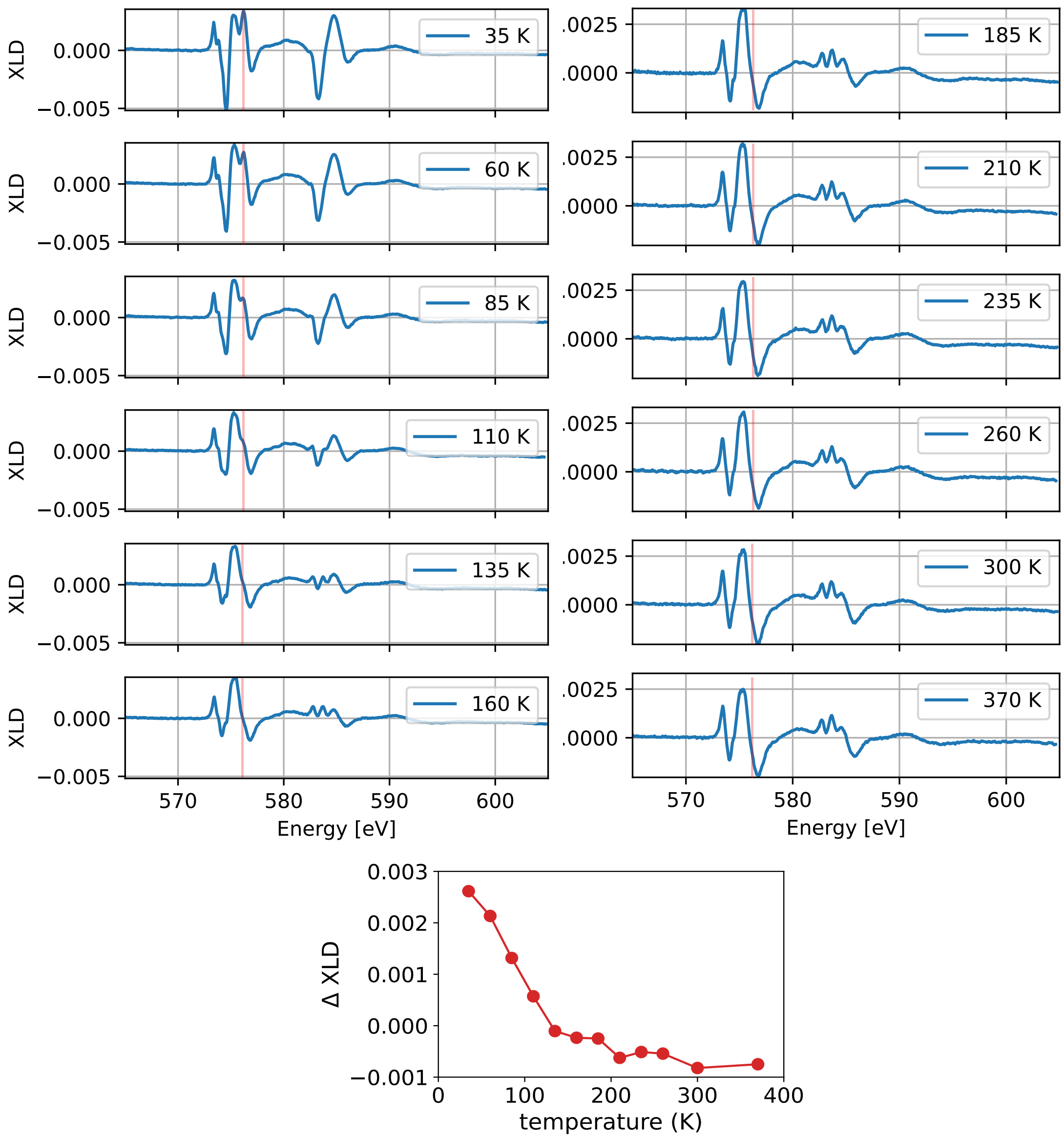

Figure S7. XLD spectra of single-layer Cr_2S_3-2D as a function of temperature, acquired with the GI X-ray beam at 6 T magnetic field. The XLD signal at 576 eV (L_3 edge) is maximal at the lowest temperature and gradually decreases as the temperature approaches the Néel temperature (T_N), becoming constant (vanishing) above. The XLD signal decrease below T_N is assigned to the alignment of the Néel vector to the in-plane magnetic field in GI orientation.

Supplementary Note 8. Location independence hysteresis behavior of dI/dV signal

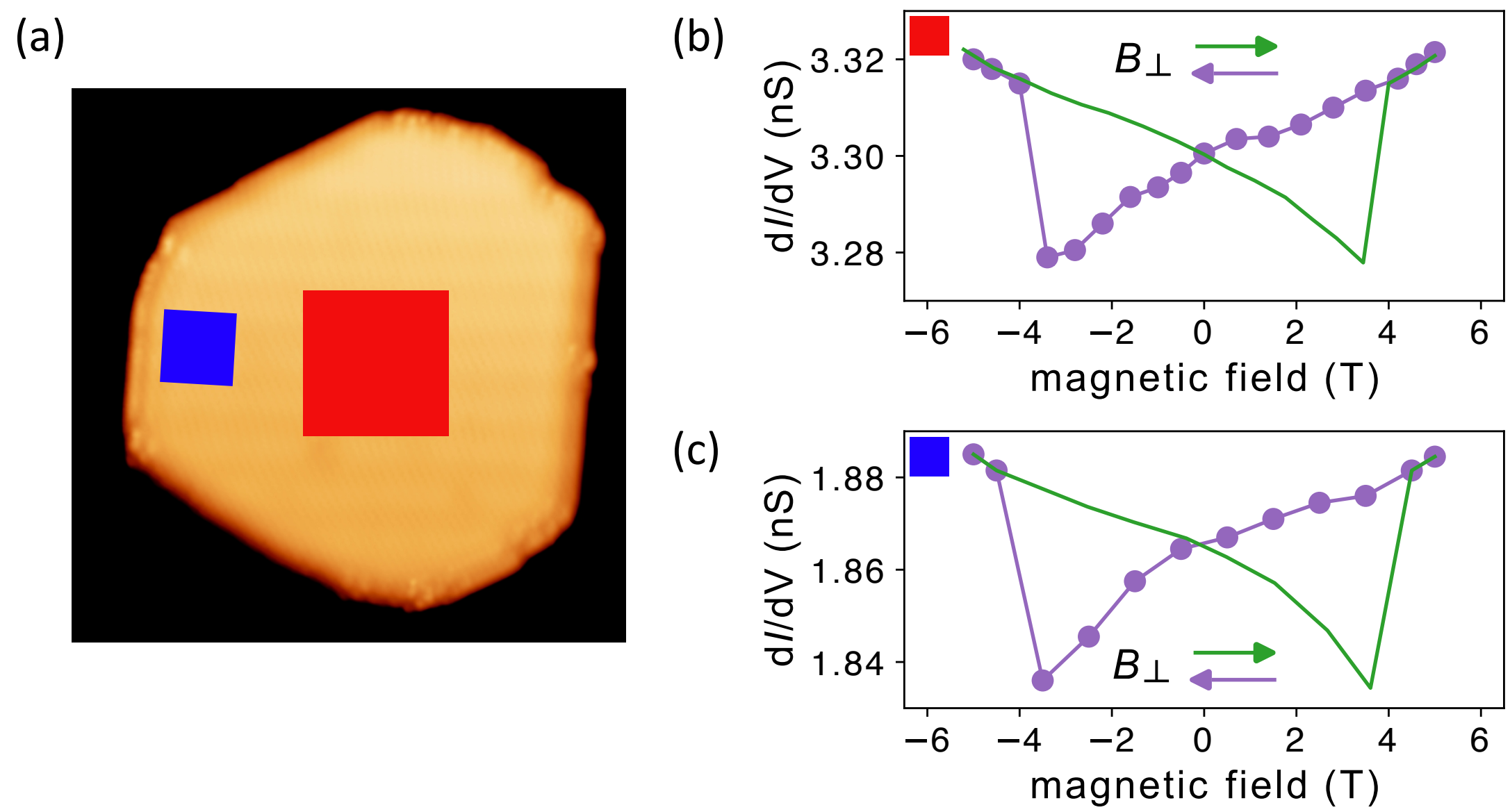

Figure S8. (a) STM overview image ($V_\mathrm{b} = 1\,\mathrm{V}$, $I_\mathrm{t} = 50\,\mathrm{pA}$, $38\,\mathrm{nm} \times 38\,\mathrm{nm}$) of a Cr_2S_3-2D island on Gr/Ir(110). The red and blue box in (a) indicate the locations of the dI/dV map acquisition ($V_\mathrm{b} = 100\,\mathrm{mV}$, $I_\mathrm{t} = 1\,\mathrm{nA}$) used for the d$I$/d$V(B)$ curves in (b) and (c). The switching fields are independent of the location.

Supplementary Note 9. Dependence of switching field on tunneling current

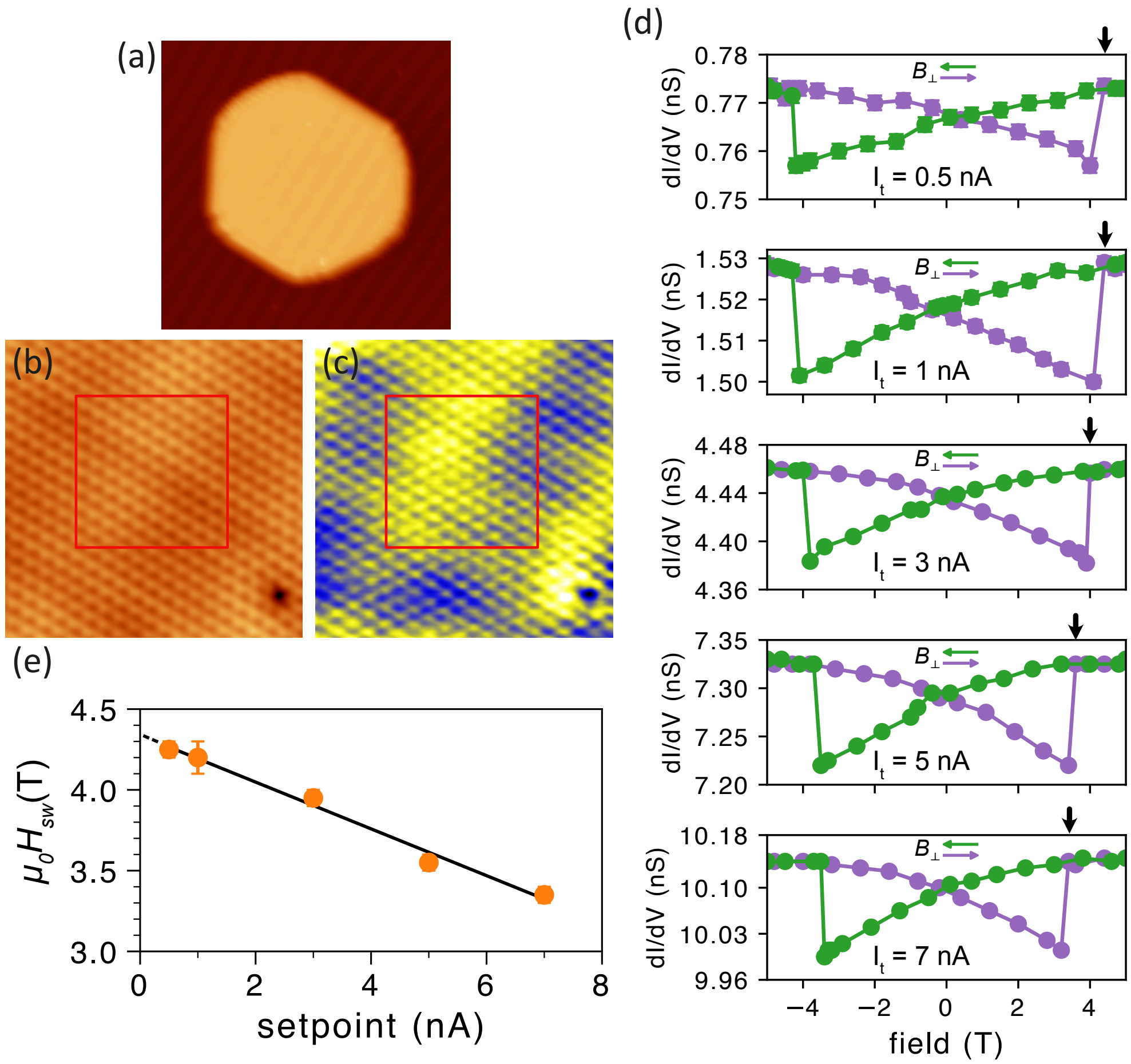

Figure S9. (a) STM overview image ($V_b = 1\,\mathrm{V}$, $I_t = 50\,\mathrm{pA}$, $50\,\mathrm{nm} \times 50\,\mathrm{nm}$) of the Cr_2S_3-2D island on Gr/Ir(110) substrate. (b) Atomically resolved SP-STM topograph ($V_b = 100\,\mathrm{mV}$, $I_t = 1\,\mathrm{nA}$, $6.5\,\mathrm{nm} \times 6.5\,\mathrm{nm}$) of Cr_2S_3-2D recorded with an out-of-plane field of $B = 1\,\mathrm{T}$. (c) $\mathrm{d}I/\mathrm{d}V$ map of Cr_2S_3-2D recorded simultaneously with (b). (d) Plot of average $\mathrm{d}I/\mathrm{d}V$ value as a function of magnetic field at $V_b = 100\,\mathrm{mV}$ and I_t as written. Each data point in the plots is taken by averaging the $\mathrm{d}I/\mathrm{d}V$ values from the $\mathrm{d}I/\mathrm{d}V$ maps taken at magnetic fields sweeping between $\pm 6.5\,\mathrm{T}$. The red box in (c) shows the area which is used for averaging the $\mathrm{d}I/\mathrm{d}V$. (e) Switching field as a function of tunneling current. The red box in (b) indicates the area used for averaging the $\mathrm{d}I/\mathrm{d}V$ signal. A linear fit of the data (black line) to zero tunneling current results in a switching field of 4.35 T, just slightly larger than the 4.2 T obtained for the standard current of 1 nA. The standard current of 1 nA was used throughout for the switching data represent in the main manuscript.

Supplementary Note 10. Temperature-dependent SP-STM of Cr_2S_3-2D.

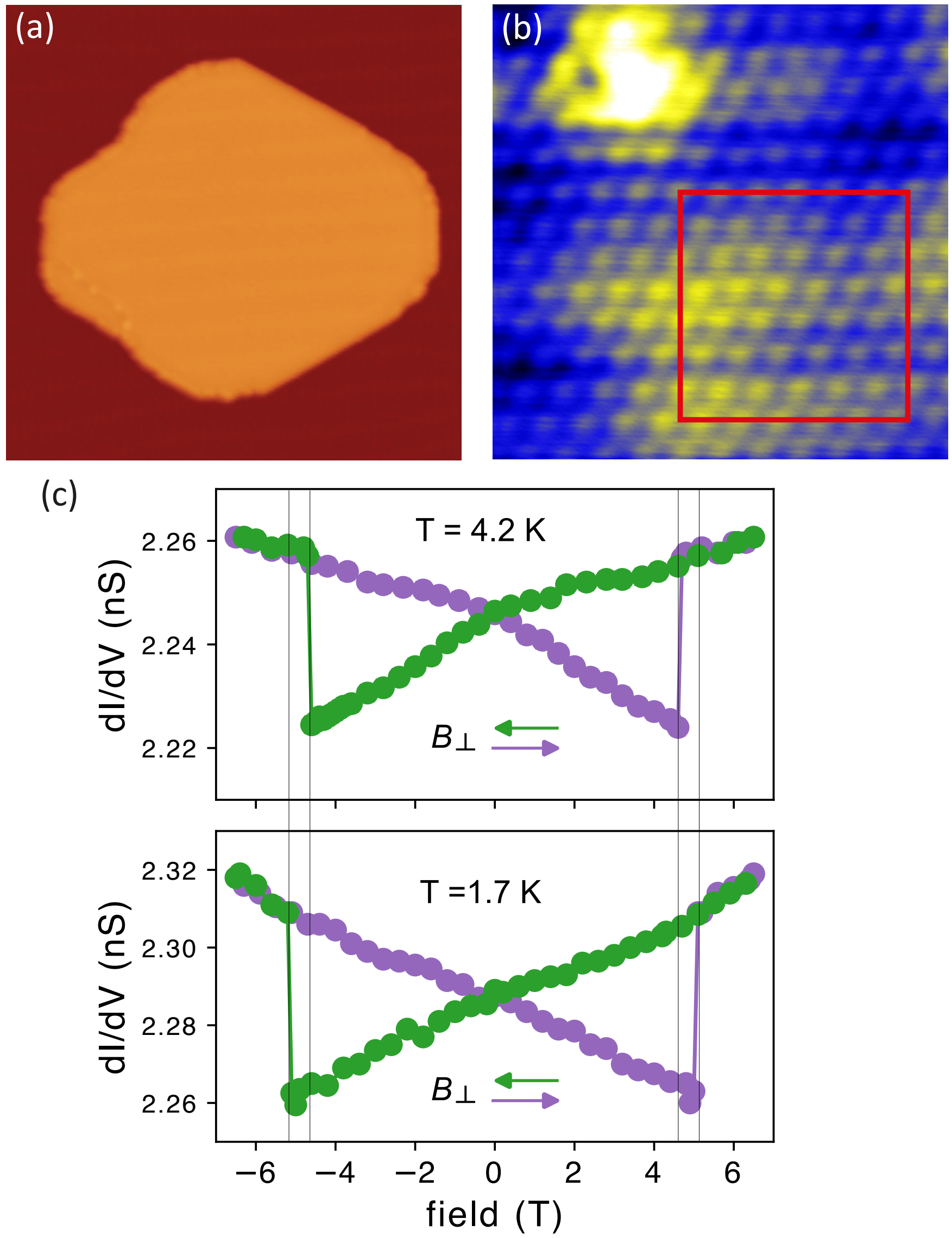

Figure S10. (a) STM overview image ($V_b = 1\,V$, $I_t = 50\,pA$, $45\,nm \times 45\,nm$) of a Cr_2S_3-2D island on Gr/Ir(110) substrate. Constant current dI/dV map ($V_b = 100\,mV$, $I_t = 1\,nA$, $4\,nm \times 4\,nm$) recorded on the Cr_2S_3-2D island shown in (a). (c) Temperature-dependent average dI/dV value as a function of magnetic field. The average dI/dV values are extracted from dI/dV maps recorded at magnetic fields between $\pm 6.5\,T$. The red box in (b) indicates the area used for averaging the dI/dV signal.

Supplementary Note 11. Energy barrier analysis with uncompensated moments arising from edges

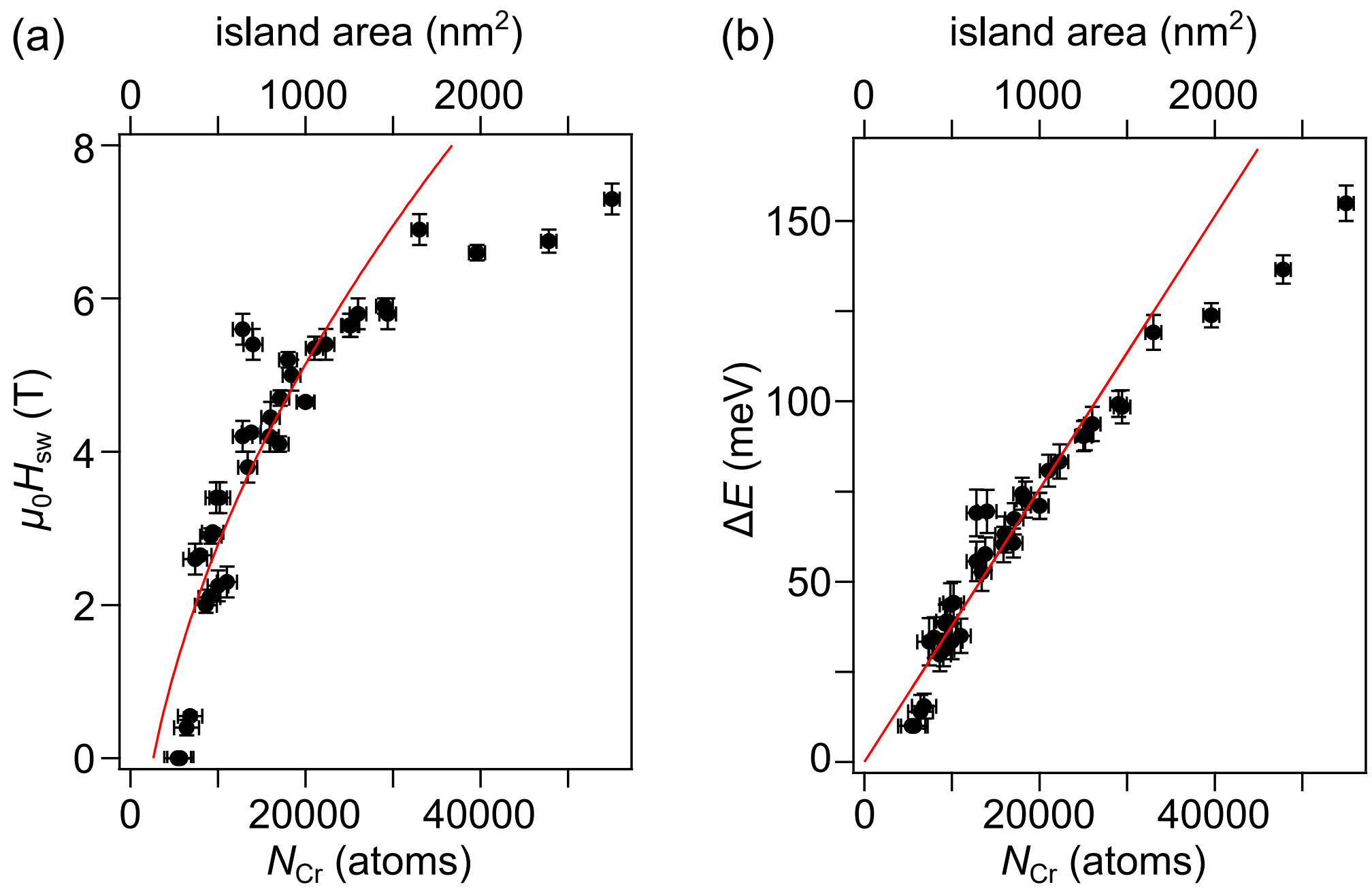

Figure S11. (a) Switching field $\mu_0 H_{\mathrm{sw}}$ and (b) energy barrier (ΔE) of islands as a function of island size. ΔE is extracted from $\mu_0 H_{\mathrm{sw}}$ in (a) by using equation 1 (main text). Red lines in (a) and (b) are fits based on equation 1 (main text) assuming the uncompensated magnetic moment to stem from island edge Cr atoms. To compute the number of Cr atoms at the edge, we assume a circular perimeter, giving $N_{\mathrm{Cr}}^{\mathrm{edge}} = \sqrt{\sqrt{3}\pi N_{\mathrm{Cr}}^{\mathrm{tot}}}$. Consequently, the total uncompensated moment is approximated as $\mu_u = N_{\mathrm{Cr}}^{\mathrm{edge}} \mu_B$.

Supplementary Note 12. DFT supercell Cr_2S_3-2D / Gr and comparison to free-standing Cr_2S_3-2D

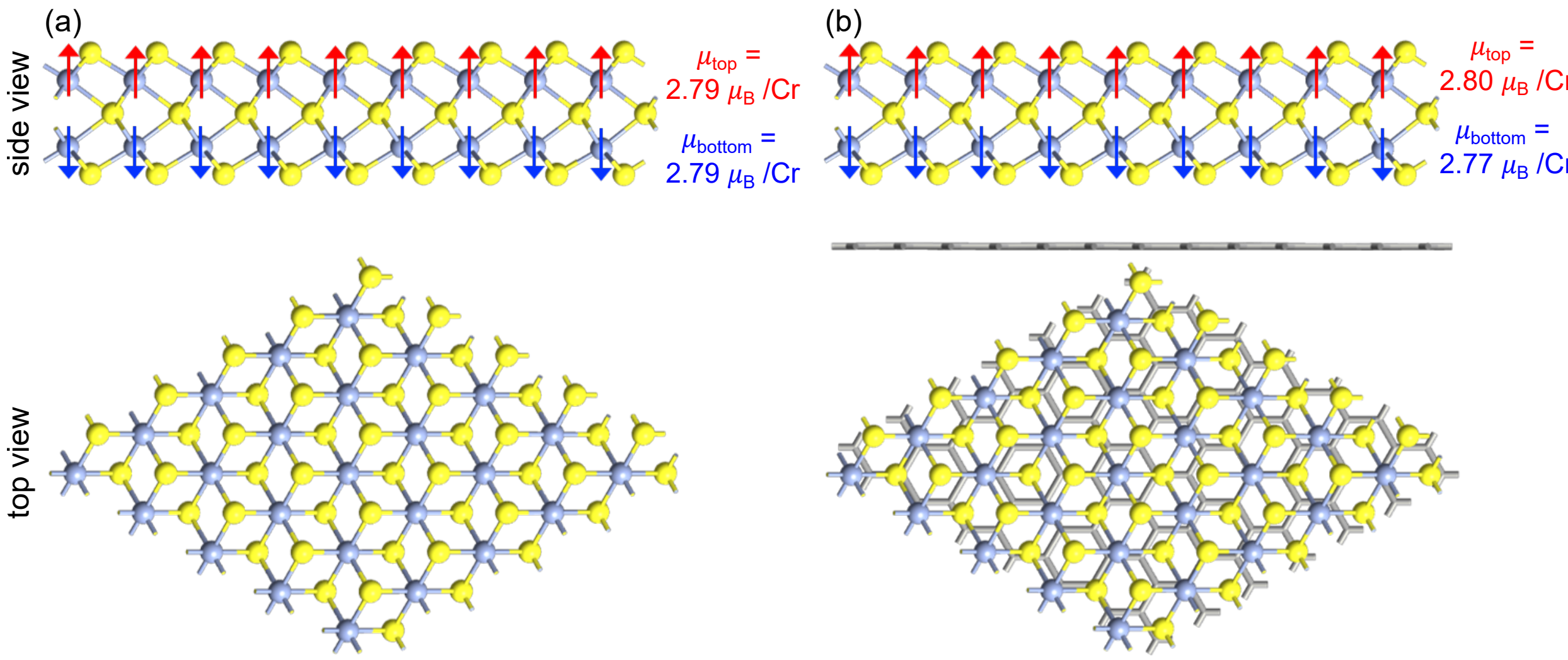

Figure S12. Side and top view ball models of the Cr_2S_3-2D freestanding (a) and supercell composed of 5 × 5 Cr_2S_3-2D layer on a 7 × 7 graphene (b). In this configuration the two layers are nearly commensurate, with strain applied to Gr of 0.23%. The DFT calculated magnetic moments of the top Cr plane μ_{top} and bottom Cr plane μ_{bottom} are given beside the side view ball model for each case. The values are equal for freestanding and differ by 0.03 μ_B/Cr for the Cr_2S_3-2D/Gr.

[1] F. Jellinek, The structures of the chromium sulphides, Acta Crystallogr. **10**, 620 (1957).

[2] T. Popma, C. Haas, and B. Van Laar, Spin structure and magnetic anisotropy of Cr_5S_6 and rhombohedral Cr_2S_3, J. Phys. Chem. Sol. **32**, 581 (1971).

[3] A. Safeer, M. Ghorbani-Asl, W. Jolie, A. V. Krasheninnikov, T. Michely, and J. Fischer, Which Chromium–Sulfur Compounds Exist as 2D Material?, Adv. Funct. Mater. **35**, e00907 (2025).

[4] S.-H. Phark, J. A. Fischer, M. Corbetta, D. Sander, K. Nakamura, and J. Kirschner, Reduced-dimensionality-induced helimagnetism in iron nanoislands, Nat. Commun. **5**, 5183 (2014).
